# Suprafenacine, an Indazole-Hydrazide Agent, Targets Cancer Cells Through Microtubule Destabilization

**DOI:** 10.1371/journal.pone.0110955

**Published:** 2014-10-29

**Authors:** Bo-Hwa Choi, Souvik Chattopadhaya, Le Nguyen Thanh, Lin Feng, Quoc Toan Nguyen, Chuan Bian Lim, Amaravadhi Harikishore, Ravi Prakash Reddy Nanga, Nagakumar Bharatham, Yan Zhao, Xuewei Liu, Ho Sup Yoon

**Affiliations:** 1 Division of Structural Biology and Biochemistry, School of Biological Sciences, Nanyang Technological University, Singapore, Singapore; 2 Division of Chemistry and Biological Chemistry, School of Physical & Mathematical Sciences, Nanyang Technological University, Singapore, Singapore; 3 Department of Genetic Engineering, College of Life Sciences, Kyung Hee University, Yongin-si, Gyeonggi-do, Republic of Korea; The Hong Kong Polytechnic University, Hong Kong

## Abstract

Microtubules are a highly validated target in cancer therapy. However, the clinical development of tubulin binding agents (TBA) has been hampered by toxicity and chemoresistance issues and has necessitated the search for new TBAs. Here, we report the identification of a novel cell permeable, tubulin-destabilizing molecule - 4,5,6,7-tetrahydro-1H-indazole-3-carboxylic acid [1p-tolyl-meth-(E)-ylidene]-hydrazide (termed as Suprafenacine, SRF). SRF, identified by *in silico* screening of annotated chemical libraries, was shown to bind microtubules at the colchicine-binding site and inhibit polymerization. This led to G_2_/M cell cycle arrest and cell death via a mitochondria-mediated apoptotic pathway. Cell death was preceded by loss of mitochondrial membrane potential, JNK - mediated phosphorylation of Bcl-2 and Bad, and activation of caspase-3. Intriguingly, SRF was found to selectively inhibit cancer cell proliferation and was effective against drug-resistant cancer cells by virtue of its ability to bypass the multidrug resistance transporter P-glycoprotein. Taken together, our results suggest that SRF has potential as a chemotherapeutic agent for cancer treatment and provides an alternate scaffold for the development of improved anti-cancer agents.

## Introduction

Microtubules - long, filamentous, tube shaped polymers – mediate important roles in cellular signaling, transport of cargos, establishment of cell polarity, maintenance of cell shape, cellular migration and cell division [1], [2]. Composed of α- and β-tubulin heterodimers bound in a head-to-tail manner, microtubules are not simple equilibrium polymers; instead they are highly dynamic structures and the rapid assembly and disassembly dynamics is crucial, in large part for their cellular functions. Not surprisingly, microtubule polymerization is subject to tight spatial and temporal regulation and this is achieved at several levels including (1) transcription of different tubulin isotypes having different functions; (2) by regulating α/β - tubulin ratios and heterodimer folding (3) through various post-translational modifications of tubulin, that in turn, alters microtubule localization and/or its interaction with signaling pathways and (4) via interaction with microtubule-associated proteins (MAPs) like dynein and kinesin motor proteins, stathmin, TOG, EB1, dynactin 1, RAC1 etc [3]–[5]. Paradoxically, the same dynamic nature of microtubules also makes them exquisitely sensitive to inhibitors. By disrupting the finely tuned behavior of microtubules, tubulin-binding drugs interfere with the process of cell division and have proved to be highly effective in cancer patients [6].

Most of the microtubule-binding drugs identified so far have been isolated from either plants or marine organisms during large-scale screens of natural products. Microtubule-targeted anti-mitotic drugs are usually classified into two groups – microtubule destabilizing agents like vinca alkaloids, colchicine and microtubule-stabilizing agents like paclitaxel and docetaxel. Though the taxanes and vinca alkaloids are still administered for a wide range of cancers and are often integrated into combination chemotherapy regimens [7], [8], the current suite of tubulin-binding drugs has several drawbacks. When compared to other anticancer drugs, microtubule-binding drugs are structurally complex, chemically diverse and have low solubility. Furthermore, the active drugs occur in only minute amounts in nature and the scarcity of their natural sources has severely hampered their clinical development. Though this issue was addressed by the development of partial or total synthesis methods [9] and via metabolic engineering of pathway intermediates [10], the problem still persists where development of new microtubule-binding compounds are concerned. Another drawback is drug resistance caused by mutations and/or expression of different tubulin isotypes like βIII-tubulin. Drug resistance may also stem from the overexpression of drug-efflux pumps, including the multidrug resistance transporter P-glycoprotein (P-gp) or multidrug-resistance associated protein (MRP) [11]. Patients being administered with microtubule-binding agents tend to suffer from peripheral axonal neuropathy that limits the tolerable dose [12]. Despite these limitations, several anti-mitotic drugs with diverse binding sites on tubulin are in various stages of clinical development. The armamentarium of microtubule-targeted agents with improved pharmacodynamic and pharmacokinetic profiles, minimal neurological toxicity and broad spectrum efficacy, continues to grow [13]–[15]. Ideally, such leads should also be devoid of P-gp-mediated drug efflux and be amenable to facile chemical synthesis approaches.

In this work we report a novel compound based on the indazole scaffold, 4,5,6,7-tetrahydro-1H-indazole-3-carboxylic acid [1p-tolyl-meth-(E)-ylidene]-hydrazide (Suprafenacine or SRF), that shows potent anticancer properties. We discuss the identification of SRF using *in silico* high-throughput screening approach, its chemical optimization and elucidation of its microtubule binding mode. SRF destabilizes microtubules leading to cell cycle arrest in the G2/M phase and cell death by apoptosis. Furthermore, we show that SRF can bypass the P-glycoprotein transporter, specifically targets cancer cells and is effective against several different cancer types.

## Materials and Methods

### Chemicals and Antibodies

Nocodazole, paclitaxel (Taxol), vinblastine, and colchicine were purchased from Sigma-Aldrich (St. Louis, MO). SRF was purchased from ChemDiv while SB203580, PD98059 and SP600125 were from Calbiochem (San Diego, CA). Antibodies were obtained from the following companies: vimentin, α- and β-tubulin, JNK-1, Mdr-1, Bcl-2, pBcl-2 (T56), pBcl-2 (S70), pBcl-2 (S87) (Santa Cruz Biotechnology, Inc., Santa Cruz, CA); pp38, pERK1/2, pJNK (Cell Signaling Technology, Denver, MA); monoclonal anti P-glycoprotein (MDR) (Sigma, USA) and monoclonal actin antibodies were from BD Pharmingen (San Diego, CA). [^3^H]vinblastine (specific activity, 11.6 Ci/mmol) and streptavidin-coated yttrium silicate scintillation proximity assay (SPA) beads were purchased from GE Healthcare (Buckinghamshire, UK). [^3^H]colchicine (specific activity, 80.4 Ci/mmol) was obtained from PerkinElmer (Boston, MA, USA).

### Cell Culture

Human cancer cell lines (HeLa, MDA-MB-231, A549, HCT15, Jurkat, PC12 and SH-SY5Y) and human fibroblast cell lines (CCL-116 and WI-38) were obtained from American Type Culture Collection (ATCC, Rockville, MD, USA). SNU16, human stomach cancer cell line, was obtained from Korean Cell Line Bank (KCLB, Seoul, Korea). The human epithelial primary cell line F10, and the parental cell line for the drug-resistant mutants, KB-3-1, and multidrug-resistant line, KB-V1 were a generous gift from Dr. Peter Dröge (NTU, Singapore) and Dr. M. Gottesman (NCI, Bethesda, MD), respectively [16]. Cell lines were grown in either Dulbecco’s Modified Eagle Medium (DMEM) or RPMI 1640 containing 10% fetal bovine serum, 1% penicillin/streptomycin and maintained at 37°C in a humidified 5% CO_2_ chamber. For KB-3-1 and KB-1V, the media was supplemented with an additional 1 mM sodium pyruvate and 10 µg/mL vinblastine, respectively. All resistant lines were incubated in drug-free media prior to cellular proliferation assays.

### Cell Cycle Analysis

Cell cycle progression was monitored using DNA flow cytometry. Cells were seeded at a density of about 2×10^5^ cells/well in a 24-well plate. When monolayers were 50–70% confluent, cells were treated with drugs as indicated. After 24 h treatment, cells were trypsinized, washed with PBS and fixed in 80% ethanol for 1 h at −20°C. The fixed cells were resuspended in propidium iodide (PI) staining buffer (10 mM Tris-HCl pH 8.0, 10 mM NaCl, 50 µg/mL PI, 10 µg/L RNase A, 0.1% NP-40) for 30 min at 4°C in the dark. The DNA content was determined on a FACSCalibur System (BD Biosciences, San Jose, CA). For each analysis, 10,000 cells were counted and the percentage of cells in each phase was calculated using ModFit LT software.

### Apoptosis Assays

Apoptosis was monitored using annexin V-propidium iodide (PI) double staining method. Cells were stained using the Annexin V-FLOUS staining kit (Roche Applied Science, Indianapolis, IN) according to the manufacturer’s instructions. Cells were analyzed by a BD LSR II flow cytometer (BD Biosciences, San Jose, CA) using the 488 nm blue laser for excitation and a 505LP mirror-530/30 BP filter and 550LP mirror-575/26 BP filter combinations to detect fluorescein and PI fluorescence, respectively. For each sample, 10,000 events were collected.

### Mitochondrial Membrane Potential Measurements

Changes in the mitochondrial membrane potential was detected using the Mitochondrial Membrane Potential Detection Kit (Stratagene, Cedar Creek, TX) and based on the use of the cationic dye, 5,5′,6,6′-tetrachloro-1,1′,3,3′- tetraethylbenzimidazolylcarbocyanine iodide (commonly known as JC-1). JC-1 forms fluorescent red aggregates in mitochondria of intact cells while in apoptotic cells, collapse of the mitochondrial potential causes JC-1 to remain in the cytoplasm in its monomeric green form. Briefly, cells were treated with SRF, in the presence or absence of JNK-inhibitor SP600125, and incubated overnight. Cells were then trypsinized, washed with PBS and pelleted (400 *g*, 5 min, RT). Next, pellets (1×10^6^ cells/sample) were resuspended in 500 µl of 1 X JC-1 staining solution and incubated for 15 min at 37°C in a 5% CO_2_ humidified atmosphere. After 2X washes with assay buffer, cells were resuspended in final volume of 500 µl of assay buffer. Fluorescence intensities were measured on a BD FACSCalibur System (BD Biosciences, San Jose, CA) and the CellQuest software was used for data analysis. Loss of mitochondrial potential was measured as the ratio of the red-to-green fluorescence; compared to untreated cells, this ratio will decrease in apoptotic cells.

### Caspase Activation Assay

Caspase-3 activity was measured using the CaspACE Assay System (Promega, Madison, WI) according to the vendor’s protocol. Briefly, 2×10^5^ cells/well were seeded in a 6-well plate. When monolayers were 50–60% confluent, cells were treated with SRF in the presence or absence of the JNK inhibitor, SP600125 and incubated overnight at 37°C in a 5% CO_2_ humidified atmosphere. After cells were harvested, pellets were washed with ice-cold PBS and resuspended in Cell Lysis Buffer. Freeze-thaw method was used to lyse cells and the lysates were kept on ice for 15 min. Following clarification of the lysates by centrifugation (15, 000 *g*, 20 min, 4°C), the supernatant was used to determine Caspase-3 activity. In a 96-well plate, 2 µl (10 mM stock) of Ac-DEVD-pNA substrate was added to the reaction mixture containing cellular lysate, DTT, Caspase Assay Buffer and deionized water in a final volume of 100 µl. Plates were incubated overnight at 20–22°C for 4 h and the absorbance was measured at 405 nm using a Benchmark Plus microplate reader (Bio-Rad, Hercules). Untreated cell extracts were used as control for the experiments.

### Immunofluorescence Microscopy

For immunohistochemistry, cells were fixed with 3.7% PFA followed by incubation in a blocking solution (5% BSA in PBS) for 30 min at 4°C. After 3X PBS washes, cells were permeabilized with 0.2% Triton X-100, washed 3 X with PBS and then incubated overnight with α- or β-tubulin antibodies at 4°C. After washing, samples were incubated with AlexaFluor 488-conjugated secondary antibody (Molecular Probes, Eugene, Oregon) for 1 h at room temperature. Slides were mounted using ProLong Gold anti-fade solution (Molecular Probes, Eugene, Oregon) containing DAPI and visualized at 60X magnification on a Zeiss LSM META510 confocal laser scanning microscopy. All post acquisition image processing and analysis was done using the MetaMorph software (Molecular Devices, LLC, Sunnyvale, CA).

### *In Vitro* Tubulin Polymerization Assay

Tubulin polymerization was measured *in vitro* using the Tubulin Polymerization Assay kit (Cytoskeleton, Denver, CO). Tubulin was dissolved in Buffer 1 (80 mM PIPES, 2 mM MgCl_2_, 0.5 mM EGTA pH 6.9, 10 µM fluorescent reporter, 1 mM GTP) to a final concentration of 10 mg/ml. To assay tubulin polymerization, a mixture containing 85 µl of tubulin (final concentration 2 mg/ml), 4.4 µl GTP (stock 100 mM), 280 µl of Buffer 1 and 75 µl of Tubulin Glycerol Buffer (80 mM PIPES, 2 mM MgCl_2_, 0.5 mM EGTA, 60% glycerol, pH 6.9), was made and kept on ice. Next, 5 µl of test compound, paclitaxel, nocodazole or control buffer was aliquotted in each well of a half area 96-well, black, flat-bottomed plate (Corning Costar). The plate was warmed for 1 min in a 37°C pre-warmed plate reader. Polymerization was initiated by pipetting 50 µl of reaction mix/well and monitored by measuring the change in fluorescence (E_x_ = 360 nm; E_m_ = 420 nm). Measurement was done using SpectraFluor Plus microplate reader (TECAN GmbH, Austria). Readings were taken every minute for 1 h (total of 61 readings).

### Tubulin Competition-Binding Scintillation Proximity Assay

This assay was performed for competition binding to the colchicine and vinblastine binding sites on tubulin and carried out in a 96-well plate. Biotin-labeled tubulin (Cytoskeleton, Denver, CO) was dissolved in G-PEM buffer (80 mM PIPES pH 6.8, 2 mM MgCl_2_, 0.5 mM EGTA, 1 mM GTP and 10% glycerol) to a final concentration of 0.8–1 mg/ml. [^3^H]colchicine or [^3^H]vinblastine (final concentration of 50 nM and 100 mM, respectively), 50 µM of SRF and 1.0 µg of biotin-labeled tubulin was mixed in a final reaction volume of 100 µl of G-PEM buffer and incubated for 45 min at 37°C. Streptavidin SPA beads (80 µg/well) was added to each reaction mix and incubated for an additional 30 min at 4°C. The radioactive counts were measured using a Wallac Microbeta scintillation counter (PerkinElmer, Waltham, MA). For control reactions, SRF was omitted from the reaction mix.

### *In Vivo* Efficacy Studies

Severe combined immunodeficiency (SCID) female mice, 4–6 weeks of age, were obtained from Animal Resources Centre (Murdoch, Australia). Mice were implanted subcutaneously in the flank with 1×10^7^ HCT15 human colon adenocarcinoma cells. The treatment started when tumor size attained a 100–200 mm^3^ or larger. Animals were treated intraperitoneally (i.p.) with 20 mg/kg/dose SRF in final dosing formulation or a 0.9% saline vehicle. Compound was given to tumor-bearing mice on days 1, 4, and 7 after staging, and tumor mass [(length×width^2^)/2] was determined once a three days for 9 days.

### Statistical analysis

All experiments were independently repeated a minimum of three times. All quantitative data are presented as means ± SEM. Comparisons between two groups were analyzed via student’s *t* test, and values of *P*<0.05 were considered to be significant.

## Results

### Identification of SRF as a novel tubulin-binding small molecule

**Figure 1:**
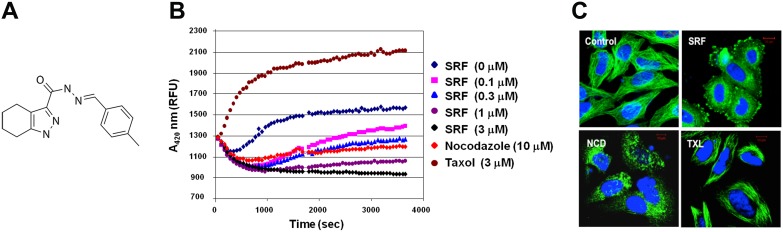
SRF - a novel tubulin-binding agent that depolymerizes microtubules. (A) Chemical structure of SRF. (B) Effect of SRF on microtubule polymerization *in vitro*. Purified tubulin was incubated at 37°C in the absence (DMSO) or presence of drugs like taxol (3 µM), nocodazole (10 µM), SRF and absorbance was measured at 420 nm every 1 min over a 60 min period. SRF inhibited microtubule polymerization in a concentration-dependent manner as was indicated by a decrease in absorbance with time. (C) Confocal micrographs of HeLa cells exposed to SRF, taxol and nocodazole. Cells were labeled with FITC-conjugated anti-tubulin antibody. SRF treatment completely destroys the intricate microtubule network. Scale bar = 10 µM.

To identify novel anticancer molecules that act via binding to tubulin, we initiated a target-based *in silico* screening of small molecules using a ChemDiv library. The initial hits identified from the virtual screen were tested for their ability to inhibit both cellular proliferation and tubulin polymerization. Of all the compounds tested, an indazole hydrazide derivative – 4,5,6,7-Tetrahydro-1H-indazole-3-carboxylic acid [1-(3-hydroxy-4-methoxy-phenyl)-meth-(E)-ylidene]-hydrazide, was found to potently inhibit microtubule polymerization (IC_50_ = 0.77 µM). To further identify analogs with improved potencies, Structure Activity Relationship (SAR) studies were carried out [Methods S1] and a focused library of 72 analogues was synthesized and screened for their ability to inhibit microtubule polymerization. This effort identified the analog 4,5,6,7-Tetrahydro-1H-indazole-3-carboxylic acid [1-*p-*tolyl-meth-(E)-ylidene]-hydrazide (analog **4**; henceforth referred to as SRF), as a potent molecule (IC_50_ = 0.38 µM) [Figure 1A].

### SRF inhibits cellular proliferation of various cancer cell types

We used a colorimetric assay to evaluate the anti-proliferative effect of SRF on cancer cells derived from a broad spectrum of representative tumor types – cervical adenocarcinoma (HeLa, KB-3-1, KB-V1), T-cell lymphoma (Jurkat), gastric carcinoma (SNU-16), breast cancer (MDA-MB-231), lung cancer (A549), colorectal adenocarcinoma (HCT-15) and neuroblastoma (SH-SY5Y) [Table 1]. All cancer cell lines tested showed susceptibility to SRF with IC_50_ values in the submicromolar range (83–381 nM). Interestingly, when compared with taxol, SRF exhibited higher potency towards the neuroblastoma SH-SY5Y and colorectal adenocarcinoma HCT-15 cell lines. Furthermore, we also tested the effect of SRF on normal cells like skin fibroblast cell line CCL-116 and human epithelial primary cell line F10. Both CCL-116 and F10 showed greater than 80% survival following a 24 h exposure to SRF while only 60% survival was seen when the same cells were treated with taxol under identical conditions [Figure S1]. Taken together, our data confirm that SRF selectively targets cancer cells and is efficacious against several different cancer types.

**Table 1:** Effect of SRF on viability of different cancer cell types.

Cell Line	Type	IC_50_	
		SRF	Paclitaxel
		nM	nM
HeLa	Cervical adenocarcinoma	200.2±8.2	6.8±0.2
Jurkat	T-cell lymphoma	210.3±2.8	5.4±0.1
SNU-16	Gastric carcinoma	381.2±28.9	2.9±0.0
MDA-MB-231	Breast adenocarcinoma	252.1±75.9	66.9±39.2
A549	Lung carcinoma	227.7±5.3	15.3±0.9
SH-SY5Y	Neuroblastoma	83.0±17.6	5241.1±365.6
HCT-15	Colorectal adenocarcinoma	237.8±5.5	437.2±70.1
KB-3-1	Cervical adenocarcinoma	206.7±3.3	6.7±0.1

### SRF can bypass the p-glycoprotein drug efflux pump

The clinical success of the drugs used in cancer treatment has been hampered by the development of drug-resistance. The phenomenon of multidrug resistance is often associated with an increase in expression of *mdr1* gene, which encodes P-glycoprotein (P-gp), an energy-dependent efflux pump. To be clinically relevant, SRF must be able to bypass the P-gp-mediated efflux. We therefore determined effect of SRF on proliferation of epidermal carcinoma cell line KB-V1, a vinblastine resistant subline derived from the parental KB-3-1 cells [16] [Table 2]. KB-V1 cells are highly enriched in P-gp and they show cross-resistance to colchicine and adriamycin [Figure S2]. Comparison of the anti-proliferative effect mediated by SRF and taxol revealed that SRF is 35-fold more potent than taxol against KB-V1 cell line while both compounds showed similar efficacy against KB-3-1 cells, as these cells are not known to overexpress P-gp [17]. The ability of SRF to bypass the P-gp probably explains why it is more potent than taxol in the SH-SY5Y neuroblastoma and HCT-15 colorectal adenocarcinoma cells as both these cancer cell types express elevated levels of P-gp protein [17]–[19].

**Table 2:** Comparison between the effect of SRF and other microtubule binding agents on viability of drug-resistant cell types.

Cell Line	Resistant Type	IC_50_			
		SRF	Paclitaxel	Colchicine	Vinblastine
		nM	nM	nM	nM
KB-3-1	Parental	206.7±3.3	6.7±0.1	18.8±0.3	0.7±0.0
KB-V1^a^	MDR↑	215.7±3.6	7692.6±151.4	777.1±36.4	256.9±42.9

a The KB-V1 is a mutant of the parental cell line KB-3-1 and resistant to vinblastine.

### SRF destabilizes microtubule assembly by binding to the colchicine-binding site

To evaluate the principal mode of action of SRF, we profiled its effect on tubulin polymerization *in vitro* [Figure 1B]. The results show that SRF inhibits tubulin polymerization in a concentration- dependent manner with an IC_50_ of 0.38 µM. In addition, the microtubule-disrupting effect of SRF was compared with two different microtubule targeted drugs, taxol, Nocodazole, known as anti-neoplastic agents by interfering the polymerization of tubulin. Notably, SRF affected the onset of the polymerization (nucleation phase) and its rate (elongation phase) resulting in reduced tubulin polymer mass. We also studied the effect of SRF on the microtubule assembly at the cellular level using immunohistochemistry. HeLa cells exposed to SRF for 24 h were fixed and microtubules stained with α- or β-tubulin antibodies. Compared with untreated control cells, which maintain an intricate and organized microtubule network, SRF treatment caused a complete destruction of this cytoskeletal network and appearance of abundant, characteristic short bundles that were distributed all over the cytoplasm in these cells [Figure 1C]. Similar disruption of the microtubules was observed with nocodazole exposure whilst taxol treatment produced shorter but denser microtubules consistent with its role as a microtubule-stabilizing agent [Figure 1C].

**Figure 2:**
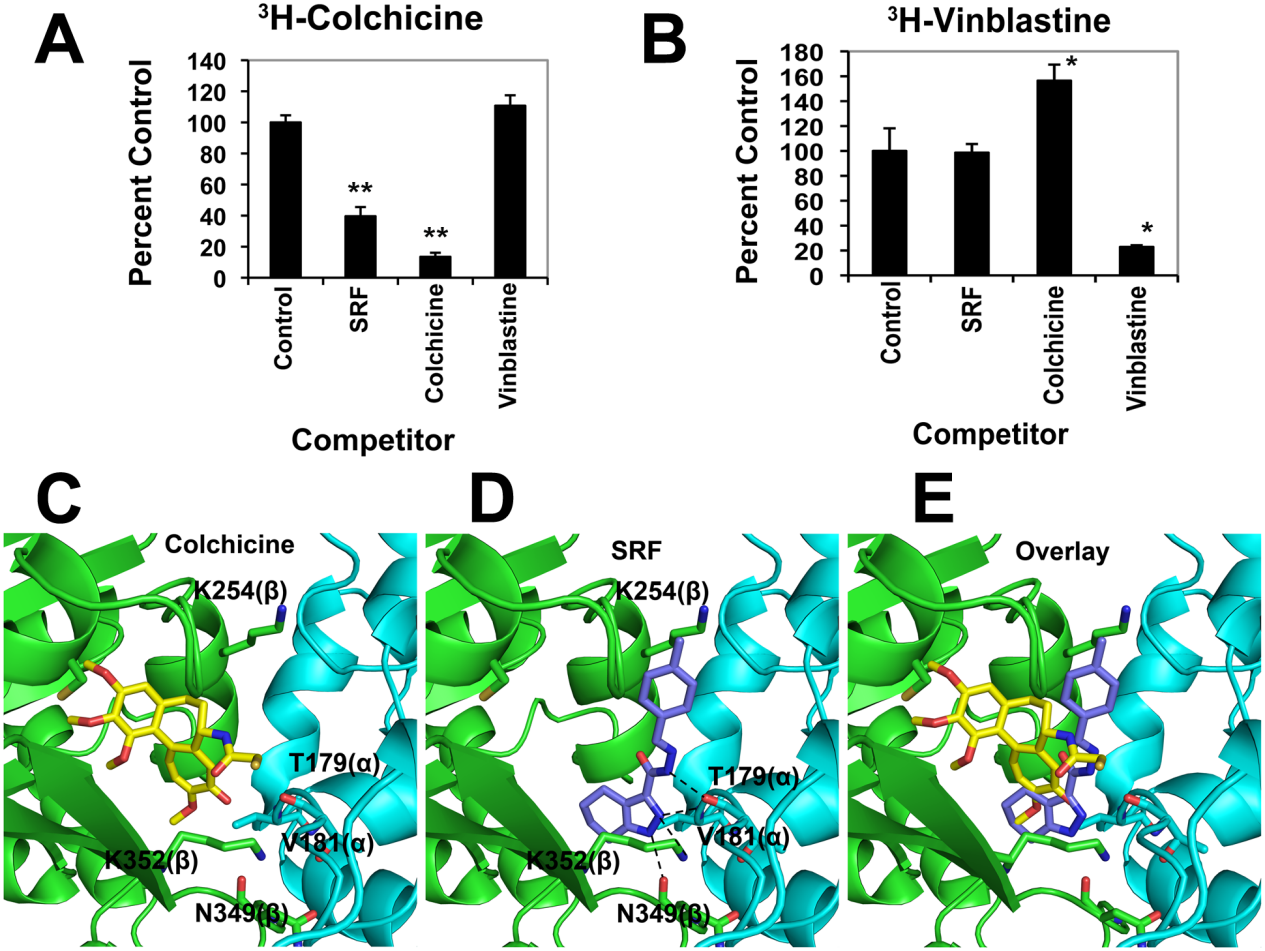
SRF binds microtubules at the colchicine-binding site. (A) SRF competes with colchicine for binding to microtubules as was determined using competition-binding scintillation proximity assay (SPA). In comparison to control (no competitor), SRF decreased colchicine binding by 2.5-fold. Data are shown as means ± SEM. *P<0.05, **P<0.01 versus control. (B) SRF and vinblastine have distinct binding sites on tubulin as SRF does not compete with vinblastine. Results shown are mean (± SD) of three independent experiments. Data are shown as means ± SEM. *P<0.05 versus control. (C–E) Cartoon representation of binding mode of colchicine (C) and SRF (D) to tubulin. The binding mode of drugs to tubulin was examined by docking studies using molecular docking program GOLD (Genetic Optimization for Ligand Docking, Cambridge Crystallographic Data Centre, UK) [Methods S1]. The compounds (colchicine or SRF) were docked into the colchicine-binding pocket of tubulin using the crystal structure of the tubulin-colchicine: stathmin-like domain [PDB code: 1SA0]. H-bond interactions between the indazole ring of SRF and N349 and K352 of β-chain (green) as well as the T179 and V181 of α-chain (blue) are highlighted. Panel E shows overlay of SRF and colchicine highlighting the difference in binding patterns of these molecules.

Compounds that inhibit tubulin polymerization mostly bind to the known distinct sites, namely taxol, vinblastine, orcolchicine sites of tubulin. A competitive-binding scintillation proximity assay (SPA) was used to probeSRF binding site on tubulin. SRF did not reduce specific SPA counts stimulated by conjugating the biotin-labeled tubulin with [^3^H] taxol or with [^3^H] vinblastine, but strongly competed for binding of [^3^H] colchicine to tubulin [Figure 2A and 2B]. Next, molecular modeling and docking studies were done to further ascertain the binding mode of SRF. Using the crystal structure of the tubulin-colchicine: stathmin-like domain (PDB 1SA0), both colchicine [Figure 2C] and SRF [Figure 2D] were docked into the colchicine-binding pocket of tubulin. Despite the structural dissimilarity, SRF shares the same cartesian space as colchicine. Unlike colchicine, SRF lacks hydrogen bond interactions with Cys241 of β-chain. Instead, the indazole ring of SRF forms hydrogen-bond interaction with Asn349, Lys352 of the β-chain as well as Val181 and Thr179 of α-chain while the 4-methyl group of the distal phenyl ring forms hydrophobic interactions with β-chain Lys 254. Taken together, the docking studies reveal that though SRF and colchicine bind to identical site on tubulin, SRF has a binding mode slightly different from that of colchicine [Figure 2E].

### SRF arrests cell cycle in G2/M-phase

**Figure 3:**
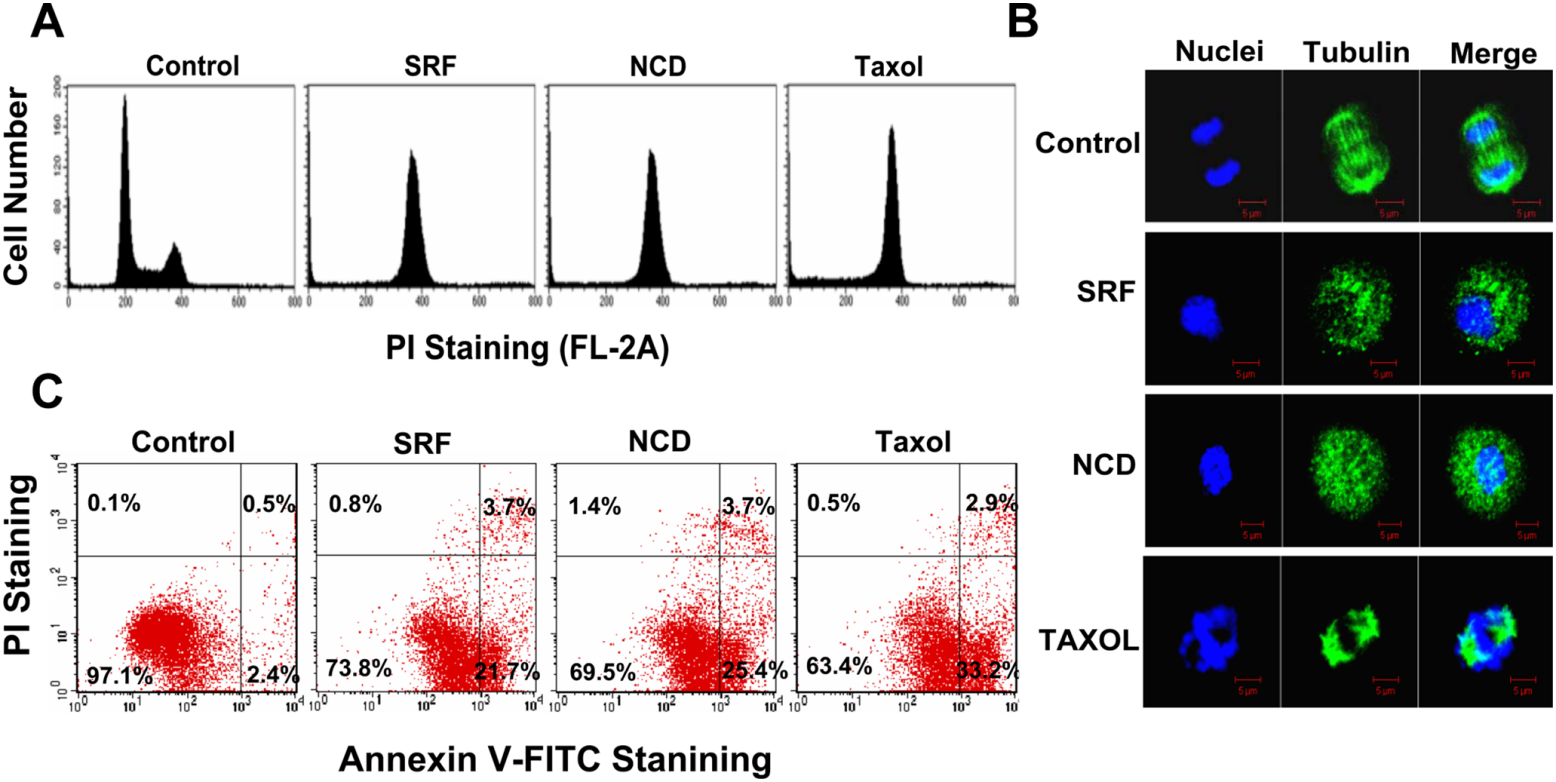
SRF-mediated microtubule depolymerization results in cell cycle arrest at G_2_/M phase. (A) Flow cytometric analysis of the DNA content of cells treated with SRF (10 µM), nocodazole or taxol. Shown are representative one-parameter histograms of treated cells. Like taxol and nocodazole, SRF-mediated inhibition of microtubule dynamics resulted in cell cycle arrest at the G_2_/M transition phase. (B) Fluorescence micrographs of mitotic cells that had been pre-treated with the indicated compounds. Compared to vehicle treated cells, SRF and other TBAs inhibited formation and segregation of the mitotic spindle. Microtubules (green) were stained with FITC-conjugated anti-tubulin antibody; nucleus (blue) was stained with DAPI. Images were acquired at 60X magnification. Scale bar = 5 µM. (C) SRF-treatment induces rapid apoptosis in proliferating cells. Following a brief 7 hr SRF exposure (10 µM), apoptotic progression was monitored by double-staining cells with Annexin V-FITC (FL-1)/PI (FL-2). During this period, percentage of annexin V^+^/PI^−^ (indicating early apoptosis) cells increased from 2.4% (in control) to 21.7%. The kinetics of this increment is similar to that seen with nocodazole and taxol. Both these compounds induced apoptosis in approximately 25.4% (nocodazole) and 33.2% (taxol) of cells within this time period. The experiments were done in triplicate (n = 3) and the data value was averaged.

Since microtubule dynamics has important roles in cell cycle progression, mitotic stall may lead to various chemotherapeutic outcomes including mitotic death, mitotic exit, apoptosis or aneuploidy [11], [20]. To study effect of SRF on cell cycle progression, HeLa cells were treated with different concentration of SRF for 24 h and cell cycle was monitored by flow cytometry. SRF treatment caused a complete collapse of all the cells at the G_1_ phase in a dose-dependent manner and dramatic increase in the G_2_/M population of the cells treated with 10 µM of SRF [Figure 3A and Figure S3]. Similar effects on microtubules were observed with nocodazole and taxol. Cell cycle arrest was accompanied by the failure of the mitotic spindle to segregate in rapidly dividing cells [Figure 3B]. However, no significant increase in the G_2_/M phase was observed in normal cell lines (CCL-116, WI-38 and F10) upon SRF treatment [Figure S4]. Since cell cycle arrest invariably triggers apoptosis, we performed annexin V-PI double staining on SRF-treated cells so as to monitor phosphatidylserine exposure, a signal for early apoptosis. Following a brief 7 h exposure, the population of early apoptotic cells (annexin V^+^/PI^−^) dramatically increased from 2.4% to 21.7% [Figure 3C], thereby indicating that SRF induces rapid apoptosis in proliferating cells.

### SRF induces apoptosis via JNK-mediated phosphorylation of Bcl-2 and Bad

**Figure 4:**
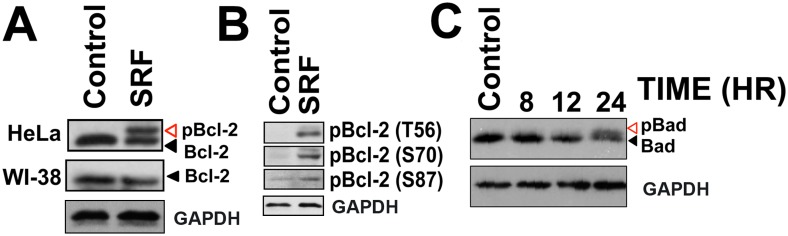
Effect of SRF on BH-3 family members, Bcl-2 and Bad. (A) Western blot analysis of HeLa and WI-38 cell extracts treated with 10 µM SRF for 24 hr and probed with anti-Bcl-2 monoclonal antibody. A slower migrating band corresponding to phosphorylated form of Bcl-2 is present in HeLa extracts but absent from WI-38 lysates, showing that SRF induces selective phosphorylation of Bcl-2 in cancer cells. (B) SRF mediates Bcl-2 hyperphosphorylation. Drug treated HeLa lysates were resolved on 12% SDS-PAGE and immunoblotting was done using specific phospho-antibodies against T56, S70 and S87 residues of Bcl-2; all these amino acids lie in the flexible loop region of the protein. (C) Time course analysis of Bad phosphorylation induced by SRF treatment. HeLa cell lysates were analyzed by immunoblotting using anti-Bad monoclonal antibody. SRF (10 µM) treatment altered phosphorylation status of pro-apoptotic protein Bad as indicated by the appearance of a slower migrating phospho-Bad band at 24 h. The band was absent from control (DMSO treated) cells even after 24 h.

The Bcl-2 family of proto-oncogenes is master regulators of apoptosis and function as molecular rheostats to control cellular survival [21]. Since anti-mitotic drugs like paclitaxel, vincristine, vinblastine, induce Bcl-2 hyperphosphorylation in the loop region [22]–[26], we investigated the effect of SRF on Bcl-2. Extracts from HeLa cells treated with 10 µM SRF for 24 h were analyzed by immunoblotting with monoclonal anti-Bcl-2 antibody. The results show that together with a Bcl-2, SRF-treated cells have additional band(s) that has slower migration rate compared to Bcl-2 [Figure 4A, Upper Panel]. In contrast to HeLa cells, vehicle-treated cells completely lacked the extra band. Likewise, the phosphorylation state of Bcl-2 in the normal diploid lung fibroblast cell line WI-38 remained unchanged even after 24 h SRF treatment [Figure 4A, Middle Panel]. To confirm that the band(s) are indeed phosphorylated forms of Bcl-2, blots were then probed with phospho-Bcl-2 specific antibodies. SRF induced the phosphorylation of Bcl-2 at multiple residues (T56, S70 and S87), all of which lie within a flexible loop region [Figure 4B]. Taken together, these observations further reiterate the fact that SRF selective targets cancer cells. In addition to Bcl-2, a 24 h exposure to SRF treatment also induced the phosphorylation of the pro-apoptotic Bcl-2 family member – Bad [Figure 4C] while Bcl-x_L_, Bak and Bax remained unaffected [Figure S5].

**Figure 5:**
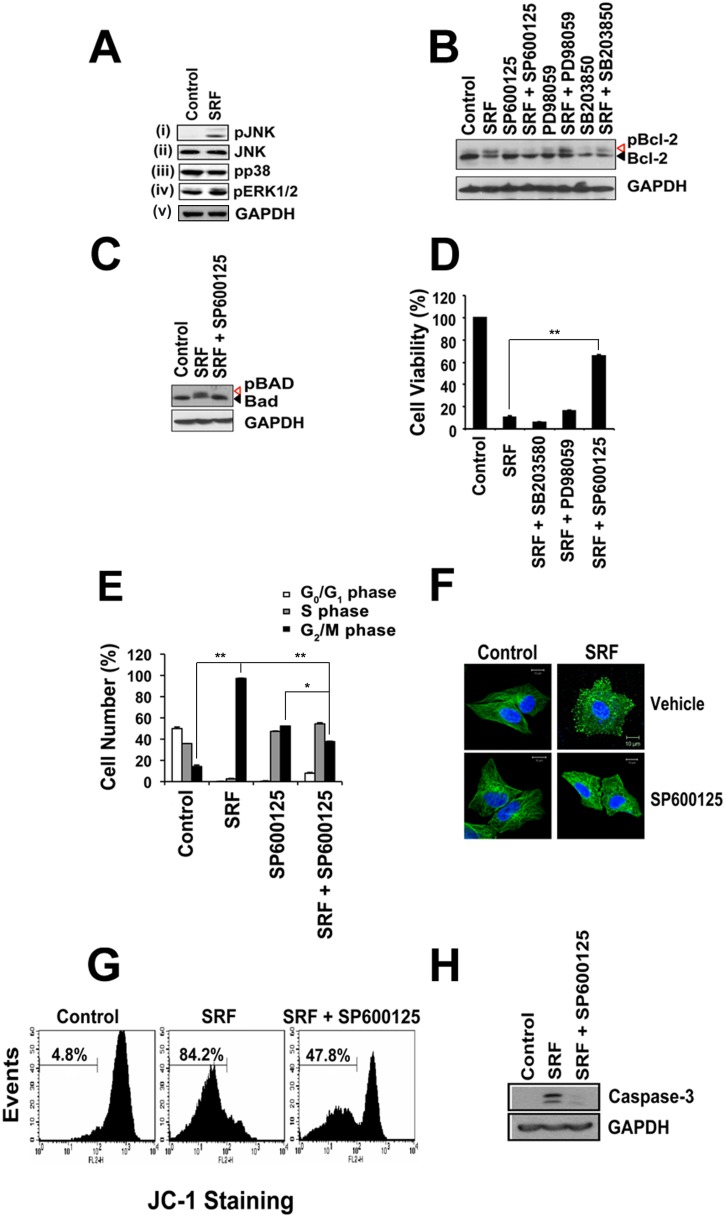
SRF cytotoxicity involves JNK kinases and proceeds via caspase-3 activation and mitochondrial membrane potential loss. (A) SRF (10 µM) activates JNK kinase but not ERK and p38. Phosphorylation status of the JNK, ERK and p38 was probed by immunoblotting using phospho-specific antibodies against the kinases. SRF selectively induces JNK phosphorylation [Panel (i)] without altering protein levels [Panel (ii)]. (B and C) Pre-treatment of cells with JNK-specific inhibitor prior to SRF (10 µM) exposure abrogates Bcl-2 and Bad phosphorylation. No phosphorylation of Bcl-2 (Panel B, Lane 4) or Bad (Panel C, Lane 3) were seen when cells were pre-treated with JNK-specific inhibitor, SP600125. Similar results were not observed with highly selective non-competitive ERK1/2 inhibitor, PD98059 (Panel B, Lane 6) or p38 inhibitor, SB203580 (Panel B, Lane 8). (D) Inhibition of JNK-kinase protects cells against SRF-induced toxicity. Cell viability was determined by MTT assay and reported as percentage control. SP600125 was able to retain viability in approximately 70% cells. Data are shown as means ± SEM. **P<0.01 versus control. (E) Cells pre-treated with SP600125 were able to overcome SRF-induced G_2_/M phase cell cycle blockage. Percentage cells in the different stages of cell cycle were determined by flow cytometric analysis. Data are shown as means ± SEM. *P<0.05; **P<0.01 versus control. (F) SP600125 treated cells retain cellular microtubule network. Fluorescence micrographs of cells treated with SRF in the presence or absence of SP600125. Microtubules (green) and nucleus (blue) were stained with FITC-conjugated anti-tubulin antibody and DAPI, respectively. Scale bar = 10 µM. (G) SRF induces loss of mitochondrial membrane potential as shown by flow-cytometric analysis of cells stained with JC-1. Events were counted in the green channel. SP600125 pre-treatment prevented cells from undergoing apoptosis as percentage of cells having fluorescence in the green channel decreased from 84.2% in SRF treated cells to 47.8% for cells that were pre-treated with SP600125 prior to SRF (10 µM) exposure. (H) Apoptotic death mediated by SRF proceeds through caspase-3 activation. A cleaved band corresponding to activated caspase-3 is present in SRF-treated lysates but absent from SP600125 pre-treated lysates.

The mitogen-activated protein kinases (MAPK), expressed in all cell types, transduce signals from the cell membrane to the nucleus in response to a variety of stimuli and also regulate a wide spectrum of biological processes critical for cellular homeostasis [27]. Among the different pathways mediated by MAPK family members, the extracellular signal-regulated kinase 1 and 2 (ERK1/2), c-Jun N-terminal kinase/stress-activated protein kinase (JNK/SAPK) and p38 MAPK, are known to be activated by cellular stresses such as heat, osmotic shock, UV and γ irradiation, metabolic poisons, pro-inflammatory cytokines and in some instances, by growth factors and GPCR agonists [28]–[31]. In fact, it has been shown that vinblastine induces apoptosis via JNK-mediated phosphorylation of Bcl-2 while all three MAPK pathways (JNK, ERK and p38 kinase) are activated upon taxol exposure [32], [33]. We therefore wanted to monitor the activation of MAPK pathway/s following treatment of HeLa cells with SRF for 24 h. Phospho-specific antibodies against JNK, ERK1/2 and p38 were used to selectively stain for activated form of these kinases. While SRF treatment had no effect on the expression levels of the kinases (data not shown), SRF induced an increase in JNK activity as evident by the appearance of a band corresponding to phosphorylated form of JNK [Figure 5A, Panel (i)]. No change in specific activity of ERK1/2 and p38 were detected [Figure 5A, Panel (iii) and (iv)]. To further verify that activation of JNK cascade is indeed casual to phosphorylation of Bcl-2 and Bad, we employed specific inhibitors - SP600125, PD98059 and SB203580 that target JNK, ERK1/2 and p38 kinase, respectively. Prior to SRF treatment, cells were incubated with the MAPK inhibitors at 10 µM concentrations for 1 h. While PD98035 and SB203580 did not alter Bcl-2 phosphorylation, pre-treatment with SP600125 completely abolished the presence of the phosphorylated forms of both Bcl-2 and Bad [Figure 5B and 5C]. To further confirm the association of the activated JNK to the hyperphosphorylation of Bcl-2 and Bad, the behavior of phosphorylated form of JNK was examined prior to 24 h post-treatment, while in parallel, the phospho-Bcl-2 and phospho-Bad were also determined after JNK inhibitor SP600125 treatment. Apparently, in the absence of SRF, none of activated form of JNK was observed [Figure S6A]. The result also confirmed the gradually decrease of phosphorylated form of JNK in a time-dependent manner (8, 12, 24 hours after SRF and SP600125 treatment) [Figure S6B]. Moreover, no phosphorylated form of Bcl-2 (no shifted band) was detected whereby about 50% decreased level of phosphorylated form of JNK was observed within 8 hours after SRF and SP600125 treatment. Taken together, our data suggest that the SRF-induced apoptosis could be mediated primarily through JNK-mediated phosphorylation of Bcl-2.

Further evidence that the JNK signaling pathway is central to SRF-mediated toxicity came from cell viability assays, immunohistochemistry and flow cytometric analysis. Pre-treatment of HeLa cells with SP600125 resulted in a 70% increase in cell viability when compared to cells exposed to only SRF while PD98059 and SB203580 treatment did not show any significant reduction in cellular toxicity [Figure 5D]. Cell cycle analysis by flow cytometry showed that 60% of SP600125 pre-treated cells can overcome the SRF-mediated G_2_/M arrest, whereas cells treated with PD98059 and SB203580 treatment continue to remain arrested in the G_2_/M phase and eventually undergoes apoptotic death [Figure 5E and Figure S6]. Furthermore, immunohistochemical analysis revealed that cells treated with SP600125 were able to maintain the intricate microtubule network that is normally destroyed upon SRF treatment [Figure 5F].

### SRF-mediated apoptosis involves via loss of mitochondrial membrane potential and activation of Caspase-3

Various extracellular and intracellular stresses the trigger the intrinsic apoptotic pathway and these signals converge mainly on the mitochondria resulting in opening of the mitochondrial transition pore (MTP), loss of mitochondrial transmembrane potential and release of cytochrome *c* together with other pro-apoptotic molecules [34], [35]. Bcl-2 family members govern mitochondrial integrity and therefore regulate these apoptotic mitochondrial events [36]. Since phosphorylation of Bcl-2 and Bad results in loss of their cellular activity, we assessed the effect of SRF on integrity of the mitochondrial membrane. Using the JC-1 reagent in flow cytometric analysis, we found that SRF treatment results in a time-dependent increase (from 4.8% to 84.2%) of the JC-1 monomeric form that eventually peaks at 24 h while pre-treatment with the SP600125 inhibitor resulted in a 10-fold increase of green fluorescence from 4.8% to 47.8% [Figure 5G]. Furthermore, loss of mitochondrial integrity was accompanied by caspase-3 activation as was evident from immunoblots with anti-caspase-3 antibody. A cleaved band corresponding to activated caspase-3 was present in extracts of cells treated with SRF while control cells completely lacked this form [Figure 5H]. Likewise, lysates from cells treated with SRF showed a 3-fold increase in caspase-3 activity over control cells when enzymatic activity was assessed using a caspase-3 specific fluorogenic tetrapeptide substrate, Ac-DEVD-pNA [Figure S6D]. In all cases, pre-treatment of cells with the JNK inhibitor SP600125 abrogated the caspase-3 activation thereby further underscoring the importance of JNK in SRF-mediated cell death pathway.

### *In vivo* efficacy of SRF against colon adenocarcinoma HCT15 xenograft

**Figure 6:**
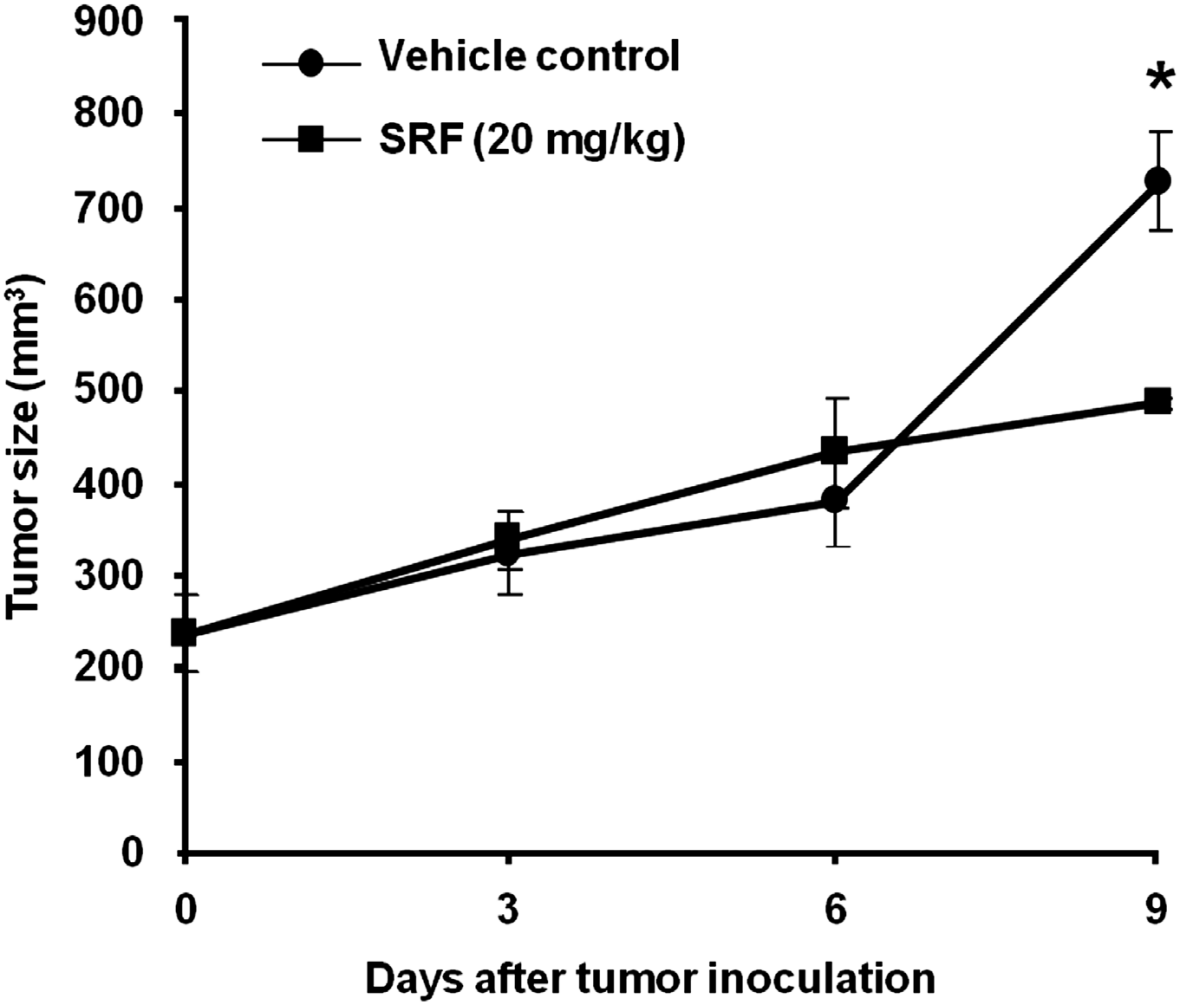
The antitumoral effect of SRF on human colon adenocarcinoma HCT15 xenografts. SCID female mice were implanted with 1×10^7^ HCT15 human colon adenocarcinoma cells. The treatment started when tumor size attained a 100–200 mm^3^ or larger. Animals were treated intraperitoneally (i.p.) with 20 mg/kg/dose SRF in final dosing formulation or a 0.9% saline vehicle. Compound was given to tumor-bearing mice on days 1, 4, and 7 after staging, and tumor mass [(length×width^2^)/2] was determined once a three days for 9 days. Three tumors were observed at different time points. Error bars represent standard error of the mean (n = 3). The data were analyzed by a one-sided Student’s *t* test, and values of P<0.05 were considered to be significant. *P<0.05 versus vehicle control.

In addition to the direct cytotoxicity of SRF to a variety of human cancer cell lines, this antitumoral effect was also examined in animal model using human tumor xenografts in mice. SRF activity retained substantially against HCT15 cells that expressed high levels of P-gp, and that were relatively resistant to taxol [Figure S2]. Thus, an anti-tumor efficacy of SRF was performed in SCID mice bearing tumors derived from HCT15 colon adenocarcinoma. 1×10^7^ HCT15 cells were implanted as s.c. at one flank per mouse [Figure 6]. Nine days after implantation, when the well-established HCT15 xenografts were obtained with tumor size about 100 mm^3^ or larger, mice were treated intraperitoneally (i.p.) with vehicle control or with 20 mg/kg/dose SRF in final dosing formulation. Compared with tumor growth in control mice, we found that SRF induced a significant reduction of tumor mass by 40% on day 9 in a time-dependent manner, suggesting that SRF potentially suppresses tumor growth *in vivo*.

## Discussion

The concept of using anti-tubulin compounds in anticancer treatment is not new. Besides inhibiting tumor cell proliferation, several of these tubulin-binding agents (TBA) can rapidly disrupt the tumor vasculature [37] and have been the mainstay of chemotherapeutic agents. However, emergence of chemoresistance and their toxic neurological side effects have hampered the clinical development of these molecules. Owing to the importance of microtubules in cell cycle progression, microtubules continue to remain as attractive target for anticancer drugs. In our effort to identify new TBAs, we performed an *in silico* virtual screening of chemical library against tubulin to identify an indazole hydrazide derivative as a novel TBA. SAR analysis and lead optimization gave us the final compound – SRF that inhibits tubulin polymerization with submicromolar potencies. Our scintillation proximity assay results show that SRF binds to the colchicine-binding site on tubulin but has a binding mode distinct from that of colchicine. Owing to its extreme toxicity, colchicine itself is not an anticancer agent but other colchicine-domain binding drugs, such as combretastatins, 2-metoxyestradiol (2-ME) and chalcones, are now being actively investigated for their anticancer activities [38]. SRF shows broad-spectrum activity as it effectively inhibits proliferation of several different cancer cell types derived from solid tumors and hematological malignancies, as well as neuroblastomas and drug-resistant [Table 1 and Table 2]. Notably, SRF is selective against cancer cells, as it had little/no effect on normal diploid cells like CCL-116, F10 and WI-38 [Figure S1 and Figure S4]. The basis of this selectivity is presently not known. SRF is not a substrate for the efflux pumps as it exhibits a similar potency irrespective of the cell’s MDR status. SRF not only exhibited its direct anti-proliferative activity in cell based assays (KB-3-1 and KB-V1), but also demonstrated an anti-tumor activity *in vivo* in human HCT15-implanted xenografts with an effective dose of SRF (20 mg/kg/day in mice) [Figure 6]. This anitumoral efficacy of SRF in animal model can be compared with other recent tubulin polymerization inhibitors, BPR0L075 and EM015, which were reported with effective doses of 50 mg/kg/day and 300 mg/kg/day in mice, respectively [13], [15]. Further studies of SRF in a variety of xenograft tumor models (for example in KB-3-1- and KB-V1-xenografted mice) might provide insightful information into chemotherapeutic potential of SRF as a novel microtubule-targeting agent. Whether SRF can overcome resistance mediated by tubulin mutations and/or expression of different tubulin isotypes also needs further investigation.

**Figure 7:**
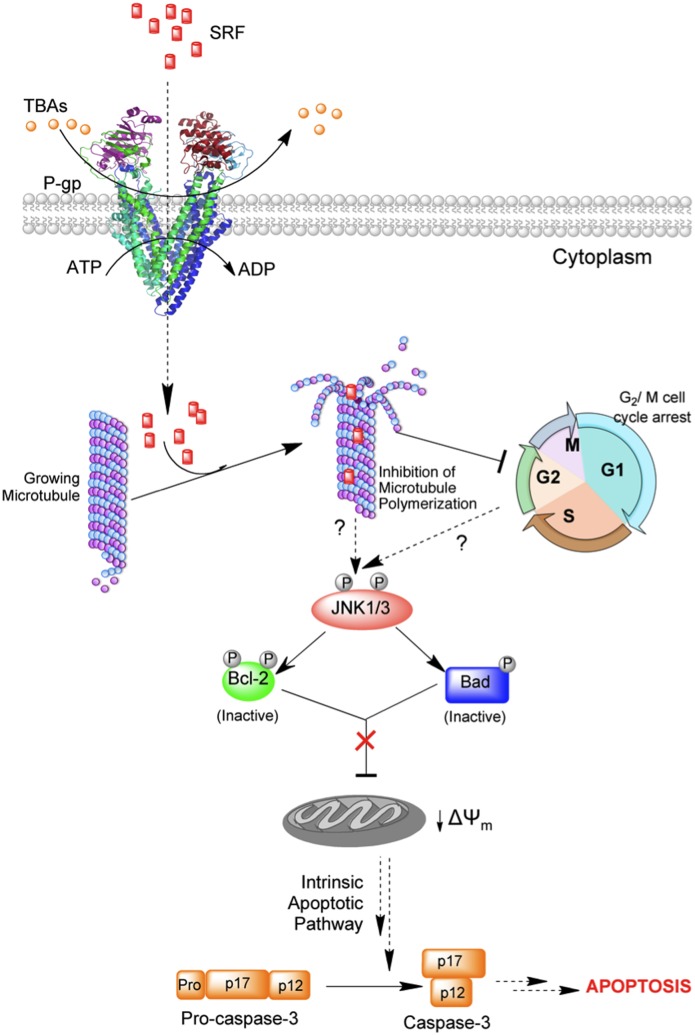
Cartoon representing plausible mechanism of SRF mediated toxicity in cancer cells. SRF can bypass P-gp mediated drug efflux via mechanism(s) that are currently unknown. Inside the cell, SRF binds and inhibits microtubule polymerization resulting in cell cycle arrest at the G_2_/M phase. These events, in turn, activate JNK-mediated stress-response signaling cascade leading to the phosphorylation and inactivation of anti-apoptotic proteins like Bcl-2 and Bad. Consequently, there is loss of mitochondrial membrane potential and integrity, release of cytochrome-c, activation of caspase-3 and eventual cell death by apoptosis. Thus, SRF-mediated cell death proceeds via the intrinsic/mitochondrial apoptotic pathway.

To further evaluate that microtubules are *bona fide* targets of SRF, the effect of compound at the cellular level was analyzed by immunofluorescence microscopy. Microtubule binding by SRF completely destroyed the cellular microtubule network causing the microtubules to bundle indicating a decrease in the polymer mass. Since attenuation of microtubule dynamics engages cell cycle surveillance mechanisms to arrest cell division in mitosis, SRF-mediated inhibition of microtubule polymerization lead to a complete blockade of the cell cycle at the G_2_/M phase and eventual cell death by apoptosis. The anti-tumor effects of SRF were shown to be associated with the microtubule depolymerization and apoptosis induction [Figure S7]. In addition, SRF-mediated cell death had all the canonical hallmarks of apoptosis as was evident from loss of mitochondrial membrane potential and concomitant activation of caspase-3. We, therefore hypothesize that SRF triggers apoptosis via the intrinsic mitochondrial pathway (Figure 7).

The Bcl-2 protein family has pivotal roles in regulating cell survival in part by affecting the mitochondrial compartmentalization of cytochrome *c* [39]. Since SRF-treatment causes loss of mitochondrial membrane integrity, we examined its effect on the Bcl-2 proteins. Bak, Bax and Bcl-x_L_ remained unaltered while Bcl-2 was hyperphosphorylated at multiple serine and threonine residues (T56, S70 and S87) that lie within the unstructured flexible-loop region of the protein. Bcl-2 phosphorylation interferes with its anti-apoptotic role as it loses its ability to heterodimerize with Bax [40], [41]. Consequently, cells arrested in the G2/M phase become increasingly sensitized to stress signals and undergo rapid apoptosis [22]. In addition to phosphor-Bcl-2, we also observed the presence of the phosphorylated form of the BH3-only protein Bad, in HeLa cell extracts treated with SRF. Bad is unique as it serves to integrate both pro-survival and proapoptotic signals with the net effect contributing to either cell survival or apoptosis. Survival-promoting kinases including Akt, Rsk, p21-activated kinase, p70S6 kinase, suppress Bad-mediated apoptosis by inducing Bad phosphorylation at S112 and/or S136 [42]–[45] and this, in turn, leads to Bad sequestration by 14-3-3 proteins [46]. On the other hand, apoptosis-inducing kinases like Cdc2 and JNK mediate Bad phosphorylation at S128 [47], [48]. Whether SRF induces Bad phosphorylation at S128 remains to be determined. Furthermore, we also delineated the signaling cascade responsible for Bcl-2 and Bad phosphorylation. Consistent with literature reports [22], [47], [49], [50] JNK was found to be involved in both cases of phosphorylation. These events were abrogated when cells were pre-treated with the JNK inhibitor SP600125 but not with PD98058 or SB203580, which inhibit ERK1/2 and p38 kinase, respectively. Cells pre-treated with SP600125 were also able to overcome G_2_/M cell cycle blockade following SRF exposure, retain mitochondrial membrane integrity and prevent caspase-3 activation thereby underscoring the importance of JNK signaling in SRF-mediated cellular toxicity.

In conclusion, the data presented herein provides compelling evidence that the novel indazole hydrazide-based compound – SRF, by virtue of (1) its has broad-spectrum efficacy against several different tumor types, (2) selectivity for cancer cell types and (3) ability to bypass multidrug resistance, has potential as an antineoplastic drug for treatment of various types of cancer. Our results warrant further investigation of the pharmacokinetic properties of SRF and determination of its *in vivo* efficacy in animal models as the SRF scaffold holds great promise as a prototype for the development of a new class of anti-tubulin agents.

## Supporting Information

Figure S1 **SRF selectively inhibits proliferation of cancer cell types.** Normal cells like skin fibroblasts CCL-116 and human primary cells F10 were treated with 10 µM of SRF for 24 hr and cell viability was measured by MTT assay. Compared to control (untreated) cells, both CCL-116 and F10 cells showed >80% survival when exposed to SRF while only 60% cells survived with taxol under identical conditions. Results shown are mean ± SEM of three independent experiments.
(PDF)

Figure S2 **Expression of P-glycoprotein on different cancer cell lines.** The total cell lysates of 11 cancer cell lines (HeLa, HCT15, MDA-MB-231, Jurkat, SNU16, SH-SY5Y, PC12, MCF-7, A549, KB-3-1 and KB-V1) were resolved by 12% SDS-PAGE and transferred into the nitrocellulose membrane for Western blot analysis. The blot was probed with monoclonal anti-P-glycoprotein antibody. GAPDH was used as a loading control.
(PDF)

Figure S3 **Concentration-dependent effects of SRF on cell cycle of HeLa cells were analyzed by flow cytometer.** Cells were treated with indicated dose-dependent concentration of SRF for 24 hours. Untreated cells were performed as negative control. Taxol-treated cells were used as a positive control. After drug treatments, cells were fixed with 80% ethanol and stained with 50 µg/mL propidium iodide for 30 min at 4°C in the dark. The DNA contents of cells were examined by flow cytometry. Results shown are from one experiment performed in triplicate.
(PDF)

Figure S4 **Effects of SRF on cell cycle progression in different human cell lines.** The percentage of the DNA content of cells treated with SRF (10 µM) was determined by flow cytometer. Shown are representative one-parameter histograms of treated cells. Unlike the cancer cell lines (MDA-MB-231, KB-V1 and A549), SRF-treated normal cell lines (CCL-116, WI-38 and F10) did not show a significant increase of cell cycle in G_2_/M transition phase.
(PDF)

Figure S5 **SRF does not phosphorylate Bcl-2 family members other than Bcl-2 and Bad.** Extracts of HeLa cells treated with 10 µM of SRF for the indicated times were probed with antibodies against Bcl-X_L_, Bak and Bax. Only a single band corresponding to the full-length protein was visible in all the blots.
(PDF)

Figure S6 **The effect of inhibition of JNK kinase on the phosphorylation of Bcl-2.** (A) SP600125 pre-treatment can prevent SRF-induced JNK phosphorylation and activation without altering protein levels. Blots were probed with phospho-JNK and JNK specific antibodies. (B) Gradual decrease in the phosphorylated form of JNK was determined in total HeLa cell lysate treated with SRF and SP600125. In correlation with changes of activated form of JNK, the phosphorylation form of Bcl-2 was detected according to the time-scale (8, 12, 24 hours post-treatment). Blots were probed with anti-phopho-JNK and anti-Bcl-2 antibodies. (C) Cells treated with p38 (SB203580) and ERK1/2 (PD98059) inhibitors are not able to overcome SRF-induced cell cycle blockade at the G_2_/M phase. Cells were stained with PI and the DNA content was analyzed by flow cytometry. (D) Caspase-3 activity in SRF-treated cell lysate was determined using the fluorogenic substrate Ac-DEVD-pNA. SRF induces a 3-fold increase in enzymatic activity that is decreased in presence of specific JNK inhibitor, SP600125. The internal protein levels were detected by using anti-histone H3 antibody.
(PDF)

Figure S7 **Molecular characterization of the anticancer effects of SRF in HeLa cells.** The cancer cells were treated with different concentrations of SRF (0, 10, 25, 50 µM) for 24 hours. Proteins (50 µg/lane) in the cell lysates were separated by SDS-PAGE and transferred to nitrocellulose membranes. The membranes were probed with anti-β-tubulin, anti-cleave PARP and anti-phosphorylated Bcl-2 antibodies. The protein expression levels of vimentin in the cell lysates was detected by anti-vimentin antibody, which is an internal loading control.
(PDF)

Methods S1 (PDF)
