# Correction: Suprafenacine, an Indazole-Hydrazide Agent, Targets Cancer Cells Through Microtubule Destabilization

**DOI:** 10.1371/journal.pone.0201149

**Published:** 2018-07-18

**Authors:** Bo-Hwa Choi, Souvik Chattopadhaya, Le Nguyen Thanh, Lin Feng, Quoc Toan Nguyen, Chuan Bian Lim, Amaravadhi Harikishore, Ravi Prakash Reddy Nanga, Nagakumar Bharatham, Yan Zhao, Xuewei Liu, Ho Sup Yoon

In Fig 5B, the label for lane 5 is swapped with the label for lane 7, and the label for lane 6 is swapped with the label for lane 8. Please see the complete, correct Fig 5 here.

**Fig 5:**
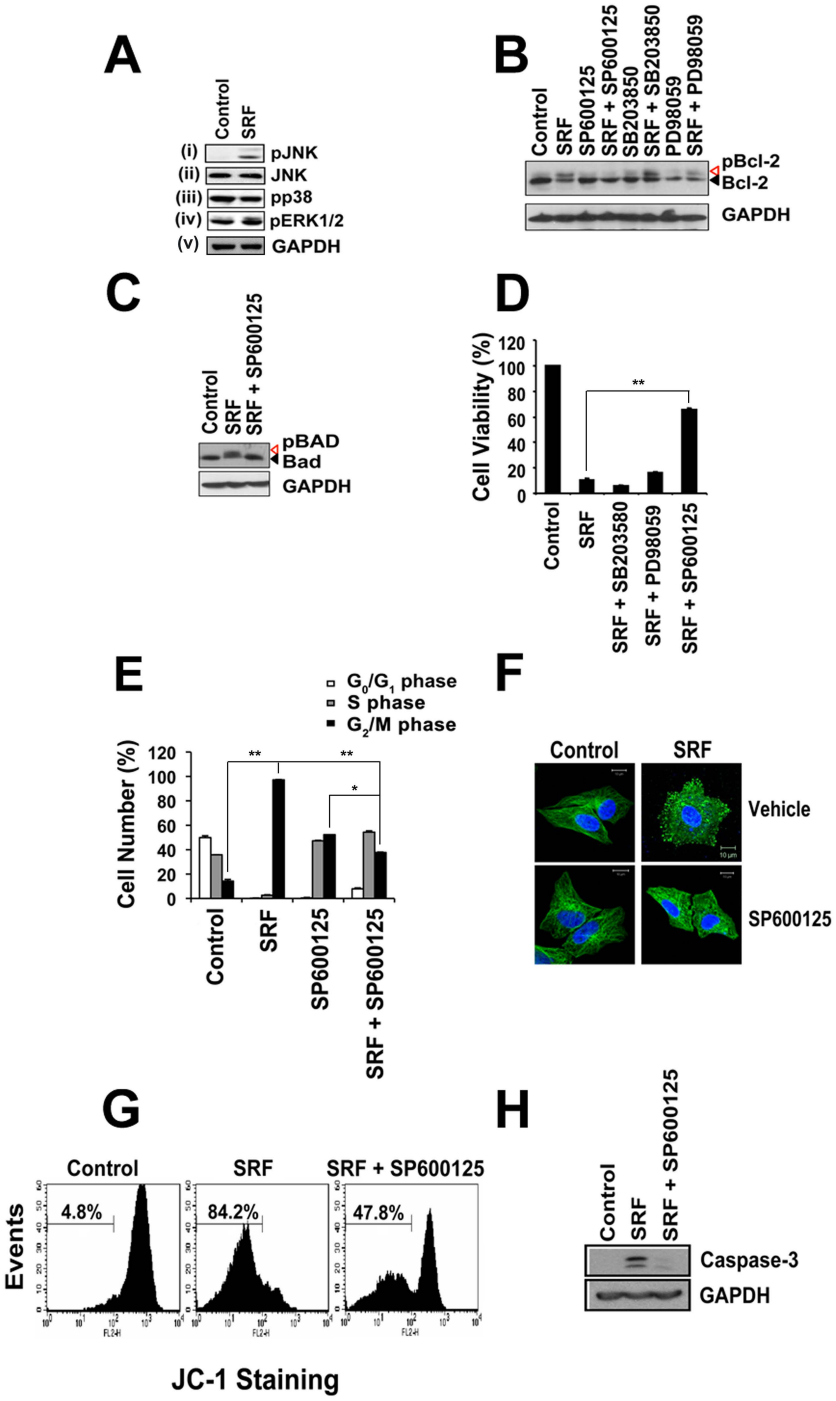
SRF cytotoxicity involves JNK kinases and proceeds via caspase-3 activation and mitochondrial membrane potential loss. (A) SRF (10 μM) activates JNK kinase but not ERK and p38. Phosphorylation status of the JNK, ERK and p38 was probed by immunoblotting using phospho-specific antibodies against the kinases. SRF selectively induces JNK phosphorylation [Panel (i)] without altering protein levels [Panel (ii)]. (B and C) Pre-treatment of cells with JNK-specific inhibitor prior to SRF (10 μM) exposure abrogates Bcl-2 and Bad phosphorylation. No phosphorylation of Bcl-2 (Panel B, Lane 4) or Bad (Panel C, Lane 3) were seen when cells were pre-treated with JNK-specific inhibitor, SP600125. Similar results were not observed with highly selective non-competitive ERK1/2 inhibitor, PD98059 (Panel B, Lane 6) or p38 inhibitor, SB203580 (Panel B, Lane 8). (D) Inhibition of JNK-kinase protects cells against SRF-induced toxicity. Cell viability was determined by MTT assay and reported as percentage control. SP600125 was able to retain viability in approximately 70% cells. Data are shown as means ± SEM. **P<0.01 versus control. (E) Cells pre-treated with SP600125 were able to overcome SRF-induced G_2_/M phase cell cycle blockage. Percentage cells in the different stages of cell cycle were determined by flow cytometric analysis. Data are shown as means ± SEM. *P<0.05; **P<0.01 versus control. (F) SP600125 treated cells retain cellular microtubule network. Fluorescence micrographs of cells treated with SRF in the presence or absence of SP600125. Microtubules (green) and nucleus (blue) were stained with FITC-conjugated anti-tubulin antibody and DAPI, respectively. Scale bar = 10 μM. (G) SRF induces loss of mitochondrial membrane potential as shown by flow-cytometric analysis of cells stained with JC-1. Events were counted in the green channel. SP600125 pre-treatment prevented cells from undergoing apoptosis as percentage of cells having fluorescence in the green channel decreased from 84.2% in SRF treated cells to 47.8% for cells that were pre-treated with SP600125 prior to SRF (10 μM) exposure. (H) Apoptotic death mediated by SRF proceeds through caspase-3 activation. A cleaved band corresponding to activated caspase-3 is present in SRF-treated lysates but absent from SP600125 pre-treated lysates.
