# Supplementary figures and images for: Suprafenacine, an Indazole-Hydrazide Agent, Targets Cancer Cells Through Microtubule Destabilization
Source: PLoS One. 2014 Oct 29;9(10):e110955. doi: 10.1371/journal.pone.0110955 (PMC4212991; doi:10.1371/journal.pone.0110955)

**Figure S1**

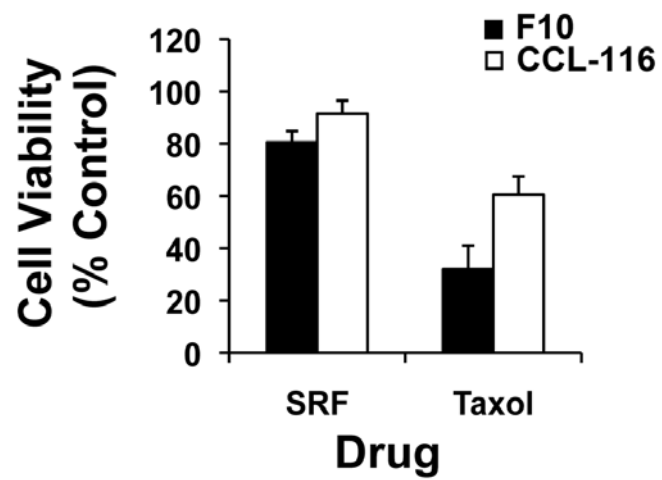

Supplement: Figure S1 — SRF selectively inhibits proliferation of cancer cell types. Normal cells like skin fibroblasts CCL-116 and human primary cells F10 were treated with 10 µM of SRF for 24 hr and cell viability was measured by MTT assay. Compared to control (untreated) cells, both CCL-116 and F10 cells showed >80% survival when exposed to SRF while only 60% cells survived with taxol under identical conditions. Results shown are mean ± SEM of three independent experiments. (PDF) [file pone.0110955.s001.pdf]

**Figure S2**

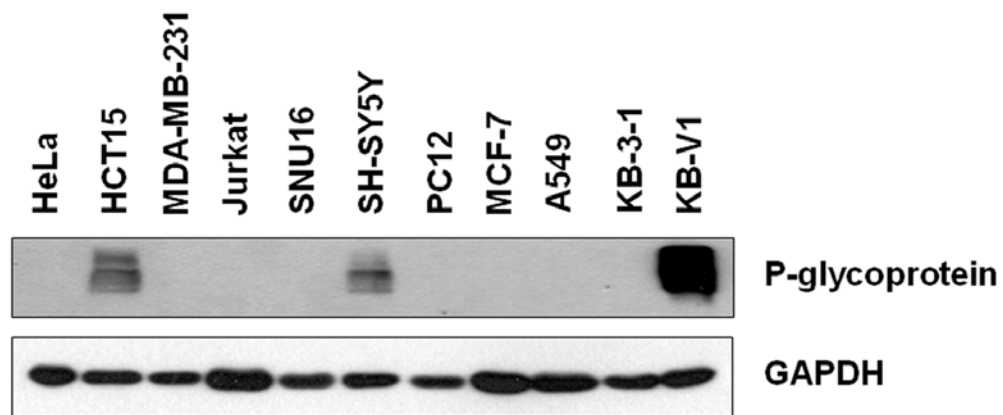

Supplement: Figure S2 — Expression of P-glycoprotein on different cancer cell lines. The total cell lysates of 11 cancer cell lines (HeLa, HCT15, MDA-MB-231, Jurkat, SNU16, SH-SY5Y, PC12, MCF-7, A549, KB-3-1 and KB-V1) were resolved by 12% SDS-PAGE and transferred into the nitrocellulose membrane for Western blot analysis. The blot was probed with monoclonal anti-P-glycoprotein antibody. GAPDH was used as a loading control. (PDF) [file pone.0110955.s002.pdf]

**Figure S3**

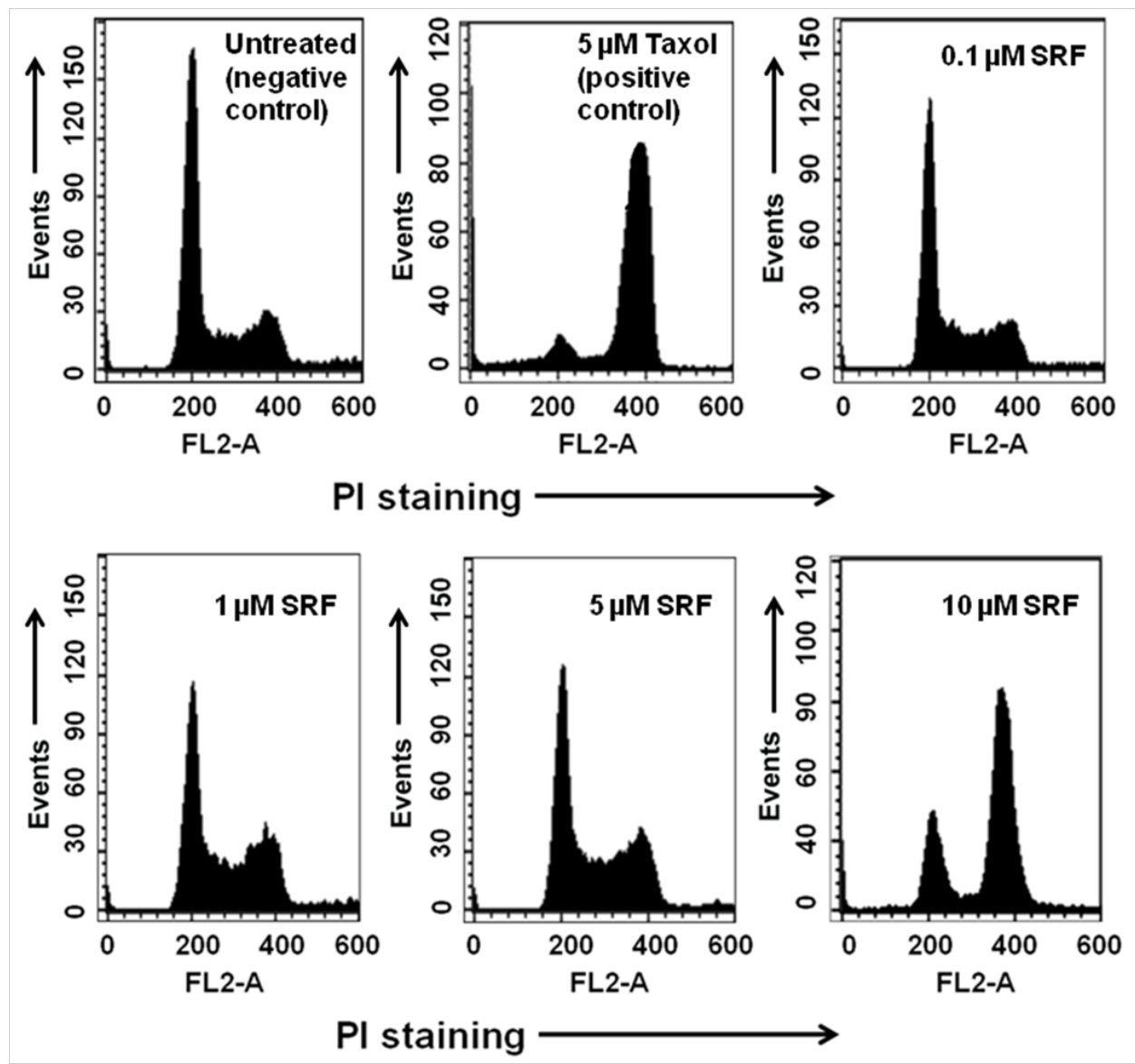

Supplement: Figure S3 — Concentration-dependent effects of SRF on cell cycle of HeLa cells were analyzed by flow cytometer. Cells were treated with indicated dose-dependent concentration of SRF for 24 hours. Untreated cells were performed as negative control. Taxol-treated cells were used as a positive control. After drug treatments, cells were fixed with 80% ethanol and stained with 50 µg/mL propidium iodide for 30 min at 4°C in the dark. The DNA contents of cells were examined by flow cytometry. Results shown are from one experiment performed in triplicate. (PDF) [file pone.0110955.s003.pdf]

**Figure S4**

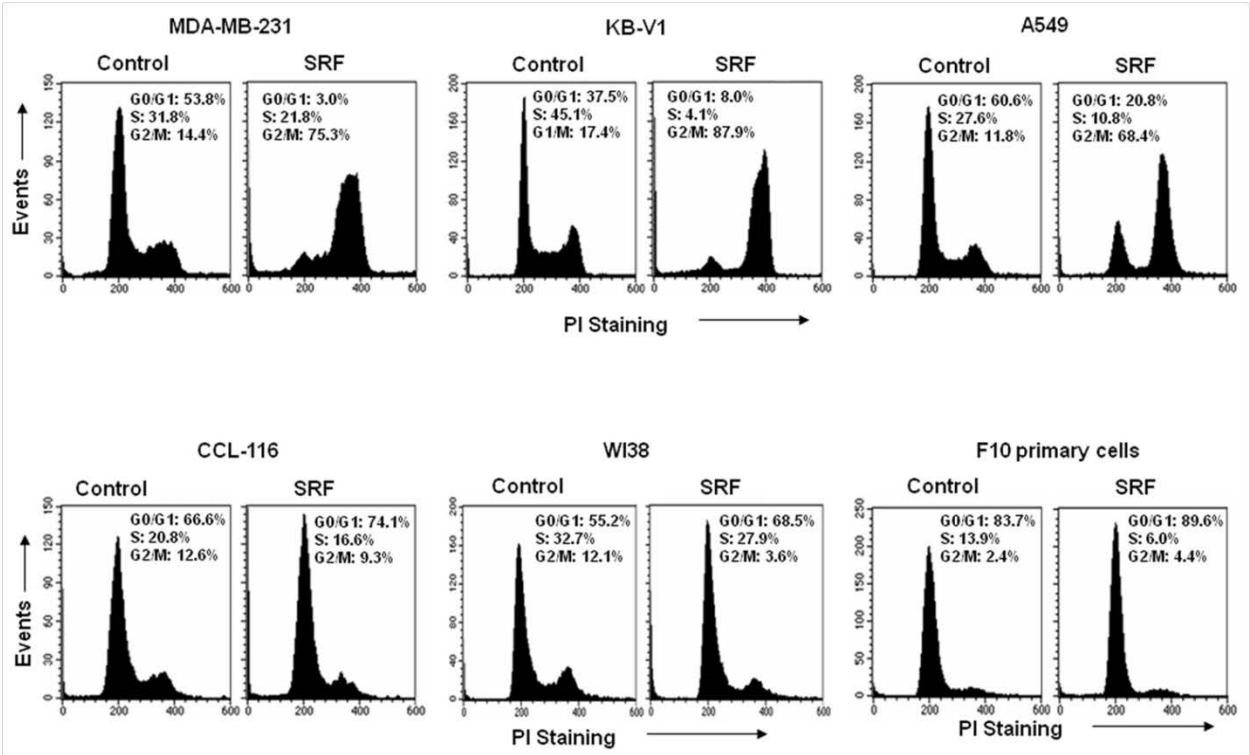

Supplement: Figure S4 — Effects of SRF on cell cycle progression in different human cell lines. The percentage of the DNA content of cells treated with SRF (10 µM) was determined by flow cytometer. Shown are representative one-parameter histograms of treated cells. Unlike the cancer cell lines (MDA-MB-231, KB-V1 and A549), SRF-treated normal cell lines (CCL-116, WI-38 and F10) did not show a significant increase of cell cycle in G2/M transition phase. (PDF) [file pone.0110955.s004.pdf]

**Figure S5**

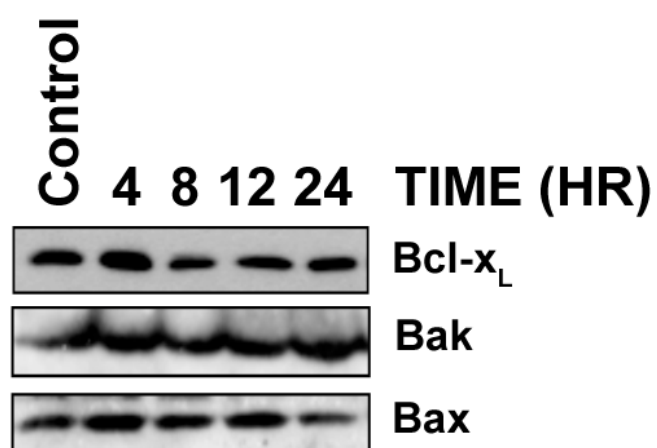

Supplement: Figure S5 — SRF does not phosphorylate Bcl-2 family members other than Bcl-2 and Bad. Extracts of HeLa cells treated with 10 µM of SRF for the indicated times were probed with antibodies against Bcl-XL, Bak and Bax. Only a single band corresponding to the full-length protein was visible in all the blots. (PDF) [file pone.0110955.s005.pdf]

**Figure S6**

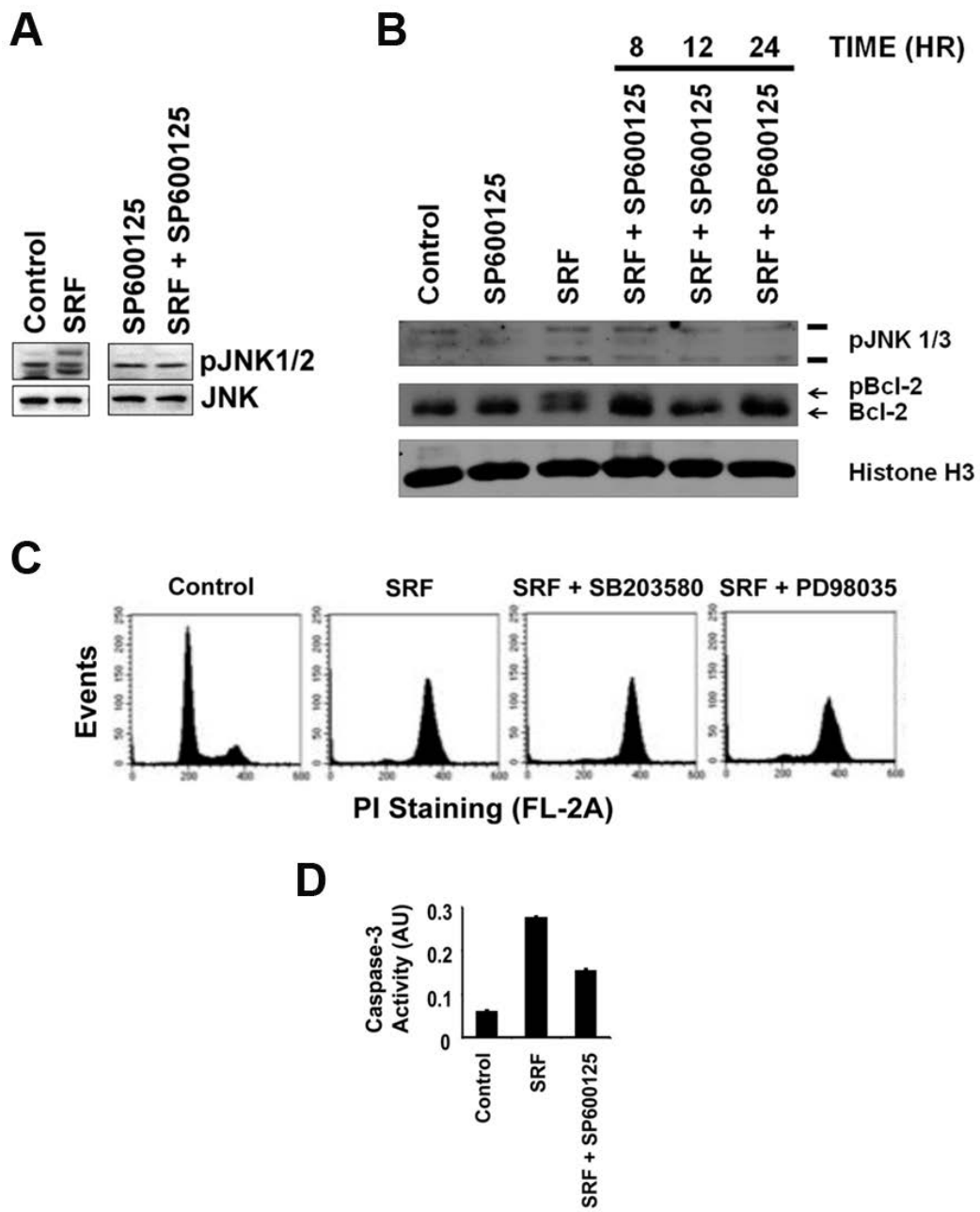

Supplement: Figure S6 — The effect of inhibition of JNK kinase on the phosphorylation of Bcl-2. (A) SP600125 pre-treatment can prevent SRF-induced JNK phosphorylation and activation without altering protein levels. Blots were probed with phospho-JNK and JNK specific antibodies. (B) Gradual decrease in the phosphorylated form of JNK was determined in total HeLa cell lysate treated with SRF and SP600125. In correlation with changes of activated form of JNK, the phosphorylation form of Bcl-2 was detected according to the time-scale (8, 12, 24 hours post-treatment). Blots were probed with anti-phopho-JNK and anti-Bcl-2 antibodies. (C) Cells treated with p38 (SB203580) and ERK1/2 (PD98059) inhibitors are not able to overcome SRF-induced cell cycle blockade at the G2/M phase. Cells were stained with PI and the DNA content was analyzed by flow cytometry. (D) Caspase-3 activity in SRF-treated cell lysate was determined using the fluorogenic substrate Ac-DEVD-pNA. SRF induces a 3-fold increase in enzymatic activity that is decreased in presence of specific JNK inhibitor, SP600125. The internal protein levels were detected by using anti-histone H3 antibody. (PDF) [file pone.0110955.s006.pdf]

**Figure S7**

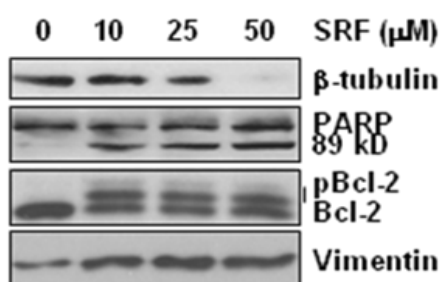

Supplement: Figure S7 — Molecular characterization of the anticancer effects of SRF in HeLa cells. The cancer cells were treated with different concentrations of SRF (0, 10, 25, 50 µM) for 24 hours. Proteins (50 µg/lane) in the cell lysates were separated by SDS-PAGE and transferred to nitrocellulose membranes. The membranes were probed with anti-β-tubulin, anti-cleave PARP and anti-phosphorylated Bcl-2 antibodies. The protein expression levels of vimentin in the cell lysates was detected by anti-vimentin antibody, which is an internal loading control. (PDF) [file pone.0110955.s007.pdf]
