# Supplementary material for: Suprafenacine, an Indazole-Hydrazide Agent, Targets Cancer Cells Through Microtubule Destabilization
Source: PLoS One. 2014 Oct 29;9(10):e110955. doi: 10.1371/journal.pone.0110955 (PMC4212991; doi:10.1371/journal.pone.0110955)
Supplement: Methods S1 — (PDF) [file pone.0110955.s008.pdf]

SUPPLEMENTARY METHODS:

Structure-Activity Relationship (SAR) analysis of SRF and its analogs

In silico screening identified the indazole derivative a indazole hydrazide derivative – 4,5,6,7-Tetrahydro-1H-indazole-3-carboxylic acid [1-(3-hydroxy-4-methoxy-phenyl)-meth-(E)-ylidene]-hydrazide (Compound **1)** as a potent microtubule inhibitor. Next structure-activity relationship (SAR) studies were done on Compound **1** to determine key features responsible for obtaining derivatives with increased potencies compared to the parent molecule.

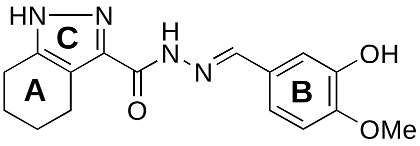

Structure of Compound **1** identified by in silico screening

Many of the analogs evaluated herein were prepared according to the procedure outlined in Scheme 1. The esters **1** were prepared in 2 steps from commercially available cyclohexanone, cyclopentanone and cycloheptanone, respectively. Ester **1** was reacted with excess hydrazine in reflux ethanol to afford hydrazide **2** in good yield. Analogues of SRF were obtained by the treatment of hydrazide **2** with substituted benzaldehydes or heterocyclic aldehydes.

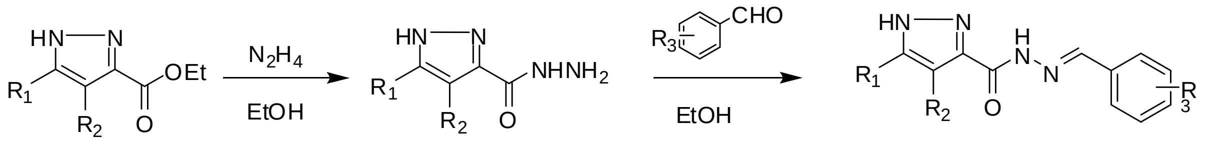

1 **2** **3**

$R_1 + R_2 = -(CH_2)_4-$

$R_1 + R_2 = -(CH_2)_3-$

$R_1 + R_2 = -(CH_2)_5-$

Scheme S1: Synthesis route for derivatives of Compound **1**

For SAR analysis, a number of substitutions were made with modifications in the rings **A**, **B** and **C** as shown in Figure 1. Ring **A** (R_1 + R_2) was substituted by 3-, 4- and 5-membered ring structures (Tables S1-3). By varying Ring **A** size, we found that compounds with 7-membered rings have decreased activity when compared to compounds that have 5- or 6 members. This indicates that the binding pocket could be small and hence does not accept bulky rings. For example, replacement of 4-membered with 5-membered ring resulted in a significant loss of activity (**1** vs. **33**, **4** vs. **39** and **13** vs. **37**) Removal of Ring **A** (**42** - **46**) also resulted in complete loss of activity. Likewise, introduction of aromaticity on Ring **A** (**47** – **49**, Table S4) failed to

show any inhibition (Taken together, our initial SAR studies reveal that only rings with 5-or 6-C long aliphatic chain are tolerated at position **A**.

Entry	$R_1 + R_2$	R_3	IC_{50} (μM)	Entry	$R_1 + R_2$	R_3	IC_{50} (μM)
1	$-(CH_2)_4-$	3-OH,4-OMe-Ph	0.77 ± 0.01	10	$-(CH_2)_4-$	3-OH-Ph	> 50
2	$-(CH_2)_4-$	3-OMe,4-OH-Ph	> 100	11	$-(CH_2)_4-$	4-OH-Ph	> 100
3	$-(CH_2)_4-$	3,4-OCH_2O-Ph	11.43 ± 5.96	12	$-(CH_2)_4-$	4-OMe-Ph	5.30 ± 1.00
4	$-(CH_2)_4-$	4-Me-Ph	0.38 ± 0.08	13	$-(CH_2)_4-$	4-Br-Ph	3.66 ± 1.03
5	$-(CH_2)_4-$	3-Cl-Ph	> 100	14	$-(CH_2)_4-$	3-pyridine	> 100
6	$-(CH_2)_4-$	2-Cl-Ph	> 100	15	$-(CH_2)_4-$	2-thiophene	>100
7	$-(CH_2)_4-$	4-Cl-Ph	5.94 ± 2.20	16	$-(CH_2)_4-$	4-COOH-Ph	>100
8	$-(CH_2)_4-$	Ph	> 100	17	$-(CH_2)_4-$	4-NO_2-Ph	>100
9	$-(CH_2)_4-$	2-OH-Ph	> 100	18	$-(CH_2)_4-$	2-OH-3,5-Bu^t-Ph	> 100

Table S1: IC_{50} values for microtubule inhibition obtained with analogs having 6C Ring **A**

Entry	$R_1 + R_2$	R_3	IC_{50} (μM)	Entry	$R_1 + R_2$	R_3	IC_{50} (μM)
19	$-(CH_2)_3-$	4-OMe-Ph	12.9 ± 1.8	26	$-(CH_2)_3-$	Ph	> 100
20	$-(CH_2)_3-$	3-OMe,4-OH-Ph	> 100	27	$-(CH_2)_3-$	2-OH-Ph	> 100
21	$-(CH_2)_3-$	3,4-OCH_2O-EvPh	26.12 ± 2.59	28	$-(CH_2)_3-$	3-OH-Ph	> 50
22	$-(CH_2)_3-$	4-Me-Ph	0.63 ± 0.01	29	$-(CH_2)_3-$	4-OH-Ph	> 100
23	$-(CH_2)_3-$	3-Cl-Ph	> 100	30	$-(CH_2)_3-$	4-NO_2-Ph	> 100
24	$-(CH_2)_3-$	2-Cl-Ph	> 100	31	$-(CH_2)_3-$	3-OH,4-OMe-Ph	0.69 ± 0.09
25	$-(CH_2)_3-$	4-Cl-Ph	1.28 ± 0.46	32	$-(CH_2)_3-$	3-OH,4-OMe-Ph	0.69 ± 0.09

Table S2: IC_{50} values for microtubule inhibition obtained with analogs having 5C Ring **A**

Entry	$R_1 + R_2$	R_3	IC_{50} (μM)	Entry	$R_1 + R_2$	R_3	IC_{50} (μM)
33	$-(CH_2)_5-$	3-OH,4-OMe-Ph	5.23 ± 0.41	38	$-(CH_2)_5-$	4-Br-Ph	61.42 ± 3.77
34	$-(CH_2)_5-$	3,4-OCH_2O-Ph	24.21 ± 0.99	39	$-(CH_2)_5-$	4-Me-Ph	5.60 ± 0.44
35	$-(CH_2)_5-$	4-Cl-Ph	> 50	40	$-(CH_2)_5-$	3-OH-Ph	> 100
36	$-(CH_2)_5-$	3-Cl-Ph	> 100	41	$-(CH_2)_5-$	2-Cl-Ph	> 100
37	$-(CH_2)_5-$	3-Br-Ph	> 100				

Table S3: IC_{50} values for microtubule inhibition obtained with analogs having 7C Ring **A**

Entry	$R_1 + R_2$	R_3	IC_{50} (μM)	Entry	$R_1 + R_2$	R_3	IC_{50} (μM)
42	$-CH_3-$	3-OH,4-OMe-Ph	>100	46	$-CH_3-$	3,4-OCH_2O-Ph	>100
43	$-CH_3-$	4-Me-Ph	>100	47	-Ph-	3-OH,4-OMe-Ph	>100
44	$-CH_3-$	4-Cl-Ph	> 100	48	-Ph-	4-Me-Ph	> 100
45	$-CH_3-$	3-OH-Ph	>100	49	-Ph-	4-Cl-Ph	>100

Table S4: IC_{50} values for microtubule inhibition obtained with analogs having non-cyclic or aromatic Ring **A**

To synthesize analogs with substitutions at Ring B, Scheme 2 was used.

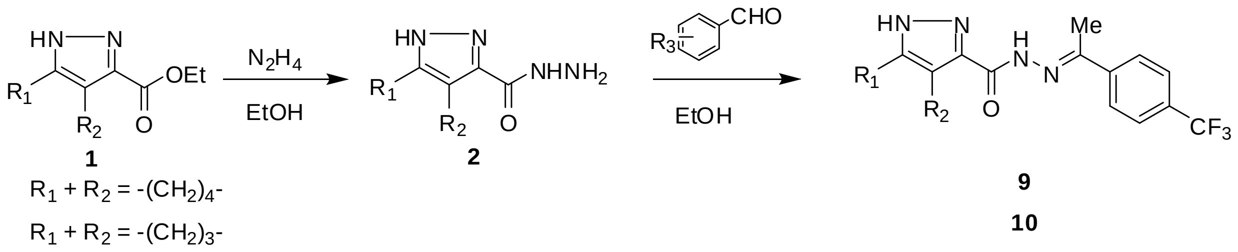

Scheme S2: Synthesis route for Ring B substituents

Next, the effect of substitution in Ring **B** was investigated (Tables 1 and 5). Analogs bearing 5- or 6C at Ring A were substituted with different R_3 groups. Our studies showed that the presence of a hydrophobic -CH_3 at 4-position of Ring **B** (**4,** Table S1) as being absolutely critical for inhibition. Removal of this invariant –CH_3 group at the 4th position (**5-8,** Table 1 and **23-30**, Table 2) or its substitution with other electron withdrawing groups like –OH (**9-11**), -COOH (**16**), nitro group (**17**) at the 2- or 3- position resulted in complete loss of activity. We also observed that bulky groups like *t*-Butyl (**50**, **58**), OCF_3 (**55**, **63**), OEt (**57**) and CF_3 (**52**, **60**) at the 4th position resulted in loss of activity.

**Entry**	**$R_1 + R_2$**	**R_3**	**IC_{50} (μM)**	**Entry**	**$R_1 + R_2$**	**R_3**	**IC_{50} (μM)**
50	-$(CH_2)_4$-	4-*t*-Bu	> 100	58	-$(CH_2)_3$-	4-*t*-Bu	> 100
51	-$(CH_2)_4$-	4-SMe	5.83 ± 0.60	59	-$(CH_2)_3$-	4-SMe	0.86 ± 0.02
52	-$(CH_2)_4$-	4-CF_3	> 50	60	-$(CH_2)_3$-	4-CF_3	5.51 ± 0.50
53	-$(CH_2)_4$-	9	> 100	61	-$(CH_2)_3$-	10	> 100
54	-$(CH_2)_4$-	NMe_2	6.99 ± 0.80	62	-$(CH_2)_3$-	NMe_2	1.32 ± 0.40
55	-$(CH_2)_4$-	OCF_3	> 100	63	-$(CH_2)_3$-	OCF_3	> 100
56	-$(CH_2)_4$-	3,4-Me_2-Ph	2.61 ± 0.80	64	-$(CH_2)_3$-	3,4-Me_2-Ph	0.68 ± 0.12
57	-$(CH_2)_4$-	4-OEt	> 100	65	-$(CH_2)_4$-	F	> 100

Table S5: IC_{50} values for microtubule inhibition obtained with substitutions on Ring B.

Finally modifications to Ring **C** were also examined by synthesizing analogs using Scheme 3.

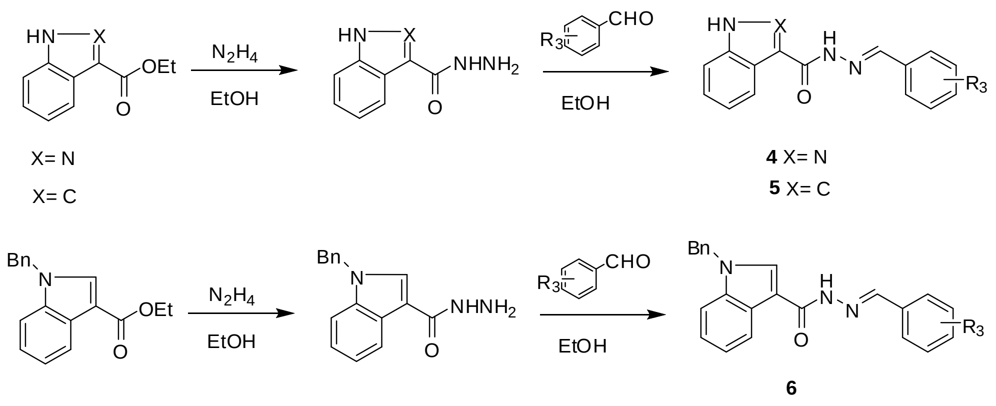

Scheme S3. Synthesis of indazole and indole compounds

As can be seen from Table S6, all the substitutions on this ring were not tolerated.

Entry	Compound	R_3	IC_{50} (μM)
66	4a	3-OH,4-OMe-Ph	> 100
67	4b	4-Me-Ph	> 100
68	4c	4-Cl	> 100
69	5a	3-OH,4-OMe-Ph	> 100
70	5b	4-Me-Ph	> 100
71	6a	3-OH,4-OMe-Ph	> 100
72	6b	4-Me-Ph	> 100

Table S6: IC_{50} values for microtubule inhibition obtained with substitutions on Ring C.

Western Blot Analysis: Following treatment, cells were scraped, centrifuged (12,000*g*, 4°C, 5 min) and cell pellets were washed with D-PBS. Pellets were re-suspended in ice-cold lysis buffer (20 mM Tris, 150 mM NaCl, 1% Triton X-100, pH 7.4) and incubated on ice for 30 min for lysis to occur. Lysates were then clarified by centrifugation at 12,000*g*, 4°C, 20 min. The supernatant

was collected and total protein concentration of the cellular extract was quantified using Bio-Rad Protein Assay kit (Bio-Rad, Hercules, CA, USA). Equal amounts of protein (20 μg/well) were then heat denatured and resolved on 12% SDS-PAGE gels. Samples were then blotted onto nitrocellulose membranes, blocked with 5% milk in TBST (25 mM Tris, 140 mM NaCl, 3 mM KCl, 0.05% Tween-20) for 1 hour at room temperature, washed and then incubated overnight at 4°C with various specific primary antibodies. Membrane where then washed with an excess of TBST and then probed with appropriate secondary antibodies. Immunoreactive bands were then visualized using Western Lightning™ enhanced chemiluminescence reagent (PerkinElmer, Boston, MA) and specific bands were visualized on CL-XPosure™ film (Thermo Scientific, Rockford, IL).

<u>Cell Proliferation Assay</u>: Cellular proliferation was determined in 96-well format using the CellTiter 96® AQ_{ueous} One Solution Cell Proliferation Assay kit (Promega, Madison, WI, USA) according to the manufacturer's instruction. This assay uses a colorimetric method to determine the number of viable cells. Briefly, 5 X 10^3 cells/well were seeded in a 96-well plate. The volume of the media in each well was 100 μl. Cells were maintained in a humidified, 5% CO_2 atmosphere till the monolayers reached 50-60% confluency (24 - 36 h). Cells were then incubated with SRF or paclitaxel for another 72 h. At the end of this period, 20 μl/well of the reagent was added to the cells, incubated for another 1-4 h and absorbance was measured at 490 nm in a 96-well plate reader. The values shown are the mean ± SD of at least three independent experiments performed in triplicates. IC_{50} were calculated based on A_{490} of untreated cells (taken as 100%).

<u>Molecular Modeling and Docking Analysis</u>:

*<u>Protein Preparation</u>***:** α- & β-tubulin sub-units of tubulin-colchicine:stathmin-like domain structure (PDB: 1SA0) was used for molecular docking studies. Charges, potentials and hydrogen atoms were assigned with CVFF force field and energy minimized for 500 steps with steepest descent and 5000 steps with conjugate gradient method in InsightII (Accelrys, San Diego, CA, USA).

Ligand Preparation: The 3D coordinates of ChemDiv library were obtained using *Prepare ligands* module and refined by applying ADMET descriptors. Next, the library was energy minimized using Smart Minimizer algorithm for 1000 steps in Discovery Studio 2.1.

Docking Analysis: Unless otherwise specified, the molecular docking program GOLD (Genetic Optimization for Ligand Docking, Cambridge Crystallographic Data Centre, UK) was used for all docking studies. The ligands were docked into the colchicine-binding site of tubulin-colchicine:stathmin like domain complex. An active site radius of 10 Å was defined around bound colchicine. Root-Mean-Square-Deviation (RMSD), annealing parameters for hydrogen bonding and van der Waals interactions were considered within 1.5, 3, and 6 Å, respectively.

General Procedure for the Synthesis of SRF and its analogs:

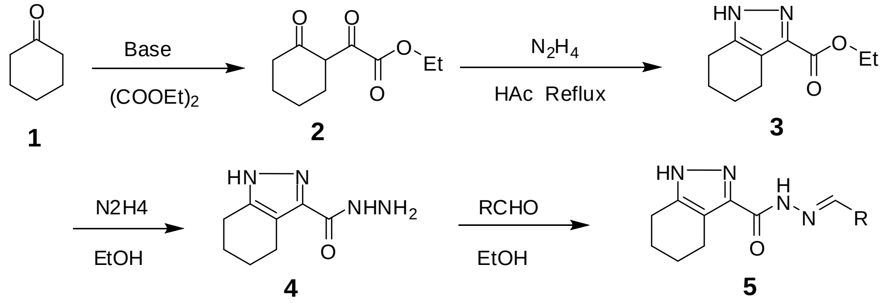

A solution of sodium ethoxide was prepared by adding sodium (1.5 g, 65 mmol) to anhydrous ethyl alcohol (20 mL) at 0°C. The mixture of cyclohexanone (4.41g, 44 mmol) and diethyl oxalate (7.3 g, 50 mmol) was then slowly added and the solution stirred for 12h at room temperature. Following decomposition of the reaction mix with 2N sulfuric acid solution, the mixture was extracted with ethyl acetate and the organic solvents were dried and concentrated. The crude product was further purified by column chromatography with n-hexane: ethyl acetate (12:1) to afford **2** (5.87 g, 67%) as a yellow oil. Next, hydrazine (448 mg, 14 mmol) was slowly added to a cooled suspension of compound **2** (2.38 g, 12 mmol) in acetic acid (5 mL). The mixture was heated to reflux for 1 h, poured into ice-H_2O, neutralized with $NaHCO_3$, and extracted with ethyl acetate. The combined organic layers were dried (Na_2SO_4), filtered, and concentrated. The residue was purified by column chromatography with n-hexane: ethyl acetate (3:1) to yield **3** (2 g, 86%) as a white solid. Ester **3** (970 mg, 5 mmol) and hydrazine (1.34 g, 35 mmol) in ethanol (10 mL) was next heated to reflux for 1day. At the end of this period, ethanol was evaporated and the precipitate collected by filtration, washed with ethyl acetate and water to

give hydrazide **4** as a white solid (580 mg, 64%). Next**,** hydrazide **4** (72 mg, 0.4 mmol) and substituted aldehydes (0.44 mmol) in ethanol (3 mL) was refluxed to obtain compound **5** as a precipitate. The precipitate was then collected by filtration and washed with cold ethanol (81%).
